# Adenovirally-Induced Polyfunctional T Cells Do Not Necessarily Recognize the Infected Target: Lessons from a Phase I Trial of the AERAS-402 Vaccine

**DOI:** 10.1038/srep36355

**Published:** 2016-11-02

**Authors:** Melissa Nyendak, Gwendolyn M. Swarbrick, Amanda Duncan, Meghan Cansler, Ervina Winata Huff, David Hokey, Tom Evans, Lewellys Barker, Gretta Blatner, Jerald Sadoff, Macaya Douoguih, Maria Grazia Pau, Deborah A. Lewinsohn, David M. Lewinsohn

**Affiliations:** 1 Department of Medicine, Oregon Health and Science University, Portland, Oregon, USA; 2 Department of Pediatrics, Oregon Health and Science University, Portland, Oregon, USA; 3 Department of Medicine, Portland VA Medical Center, Portland, Oregon, USA; 4 Aeras, Rockville, Maryland, USA; 5 Janssen Infectious Diseases and Vaccines (formerly Crucell), Leiden, The Netherlands

## Abstract

The development of a vaccine for *Mycobacterium tuberculosis* (Mtb) has been impeded by the absence of correlates of protective immunity. One correlate would be the ability of cells induced by vaccination to recognize the Mtb-infected cell. AERAS-402 is a replication-deficient serotype 35 adenovirus containing DNA expressing a fusion protein of Mtb antigens 85A, 85B and TB10.4. We undertook a phase I double-blind, randomized placebo controlled trial of vaccination with AERAS-402 following BCG. Analysis of the vaccine-induced immune response revealed strong antigen-specific polyfunctional CD4^+^ and CD8^+^ T cell responses. However, analysis of the vaccine-induced CD8^+^ T cells revealed that in many instances these cells did not recognize the Mtb-infected cell. Our findings highlight the measurement of vaccine-induced, polyfunctional T cells may not reflect the extent or degree to which these cells are capable of identifying the Mtb-infected cell and correspondingly, the value of detailed experimental medicine studies early in vaccine development.

To eradicate tuberculosis (TB), a multifaceted approach is needed, including the development of a robust and durable vaccine^1^,^2^. Whereas serologic correlates of protective immunity have been established for many vaccine preventable illnesses, correlates for protective immunity for TB have remained elusive^3^. Containment of Mtb infection requires the induction and maintenance of a robust Th1 immune response^2^,^4^,^5^,^6^ and evidence from pre-clinical animal^7^ and human^8^ vaccination studies suggest the breadth of the vaccine-induced cytokine response (IFN-γ and TNF-α, IL-2) is associated with efficacy^9^. Collectively, these T cells have been termed polyfunctional^10^. Recent results from the first Phase IIb vaccine study using MVA-Ag85A in human infants has highlighted the possibility that the induction of polyfunctional CD4^+^ T cell immunity, while important, may not be sufficient^11^ to confer protection.

While human Mtb specific CD4^+^ and CD8^+^ T cells are similar in the cytolytic and pro-inflammatory capacity^12^,^13^, CD8^+^ T cells are capable of discerning Mtb-infected cells, particularly those that are HLA-II negative. Human Mtb-specific CD8^+^ T cells are further distinguished by both their preferential recognition of heavily infected cells and restriction by HLA-B^14^,^15^. Additionally, it is increasingly evident that CD8^+^ T cells have an important and complex role in Mtb containment and immunity^14^,^16^,^17^,^18^,^19^,^20^. Specifically, we note that CD8^+^ T cells are uniquely capable of discerning the Mtb-infected cell, and that a role for these cells in the long-term progression of mycobacterial growth has been demonstrated in the mouse and non-human primate models. For most vaccination studies, the assessment of vaccine-induced CD8^+^ T cells has relied upon the measurement of antigen-specific polyfunctional cells, typically using peptide pools. However, as these measurements have been considered as a surrogate of protective immunity, it leaves open the question as to whether or not polyfunctional CD8^+^ T cells are capable of recognizing epitopes displayed in the context of Mtb infection and hence leaves open the possibility that a key parameter of vaccine immunogenicity may be overlooked.

AERAS-402 is a replication-deficient serotype 35 adenovirus containing DNA that expresses a fusion protein that includes three Mtb antigens, 85A (Ag85A), 85B (Ag85B) and TB10.4^21^,^22^. Prior work has established that AERAS-402 boosting of BCG vaccination elicits high-frequency, polyfunctional CD4^+^ and CD8^+^ T cells in adults and infants^21^,^23^,^24^,^25^. To further study human cellular immune responses to AERAS-402 and define the capacity of vaccine-induced CD8^+^ T cells to recognize Mtb-infected cells, we performed a phase I double-blind, randomized, placebo-controlled trial.

## Results

### Study enrollment, vaccine administration, and immunologic studies

Eleven adults between the ages of 18 and 45, without exposure to Mtb were enrolled (Tables 1 and 2). All received BCG vaccine 84 days prior to adenoviral vaccination. After randomization, 9 participants received AERAS-402 at day 0, 8 received AERAS-402 at day 28, and 2 received placebo at day 0 and 28 respectively (Supplementary Fig. S1; Consort Diagram^26^). To perform immunologic characterization of vaccine-induced epitopes, study participants underwent leukapheresis prior to (day -14) and after AERAS-402 vaccination (between day 56 and 98). Peripheral blood mononuclear cells (PBMC) for intracellular cytokine staining (ICS) and IFN-γ ELISPOT was collected on days −84, −14, 28, and 56 respectively (Table 3 and Supplementary Table S1). ICS was also performed on day 98. ICS and IFN-γ ELISPOT assays were performed as described previously^24^,^27^ using synthetic peptide pools with 15-mers overlapping by 11 amino acids (aa) from each antigen contained within AERAS-402. For the CD8 ELISPOT assay (CD8/others), CD8^+^ T cells were negatively selected from peripheral blood mononuclear cells (PBMC) using a combination of CD4 and CD56 magnetic beads. For the PBMC ELISPOT, unfractionated PBMC were used as the source of responding T cells and largely consist of CD4^+^ T cells.

### AERAS-402 recipients display strong antigen-specific T cell responses after vaccination

**Figure 1:**
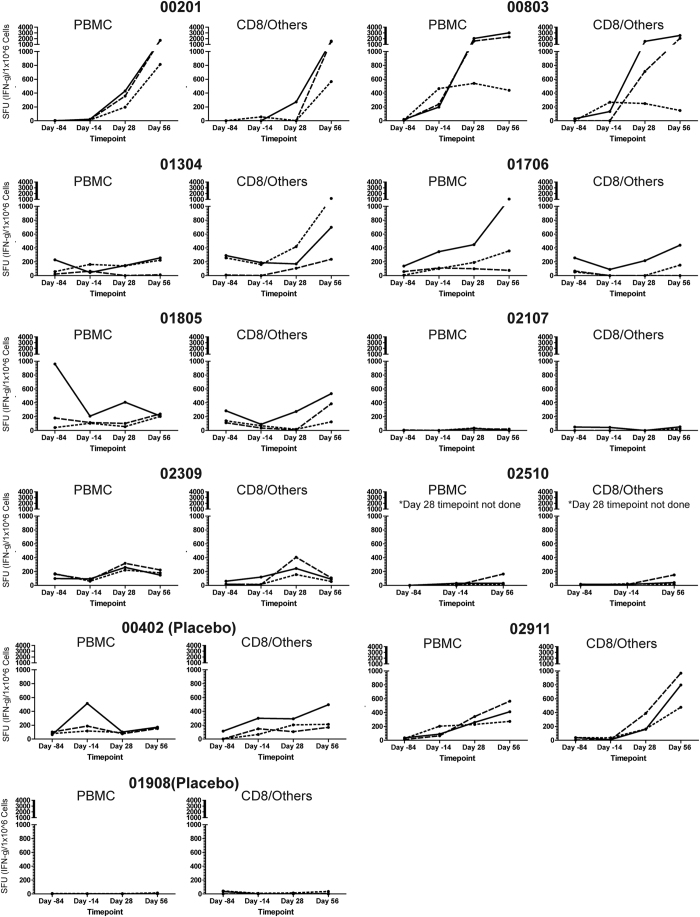
*Ex Vivo* ELISPOT Response to the AERAS-402 Vaccine. IFN-γ ELISPOT was performed on either 250,000 PBMC or 250,000 CD4-depleted PBMC (CD8/others) using single peptide pools consisting of 15-mers overlapping by 11 amino acids (final concentration of each peptide pool is 5μg/ml) spanning each of the three antigens contained in the AERAS-402 vaccine. All determinations are performed in duplicate. Solid line: Ag85A, dashed line: Ag85B, dotted line: TB10.4. For the CD8 ELISPOT assay (CD8/others), CD8^+^ T cells were negatively selected from PBMC using a combination of CD4 and CD56 magnetic beads. For the PBMC ELISPOT, unfractionated PBMC were used as the source of responding T cells and largely consist of CD4^+^ T cells. Participant numbers are designated over the graphs with the two participants receiving placebo noted in parentheses. Participant 02309 received only the first dose of AERAS-402 vaccine (day 0), due to an abnormal hematocrit prior to the planned day 28 dose.

**Figure 2:**
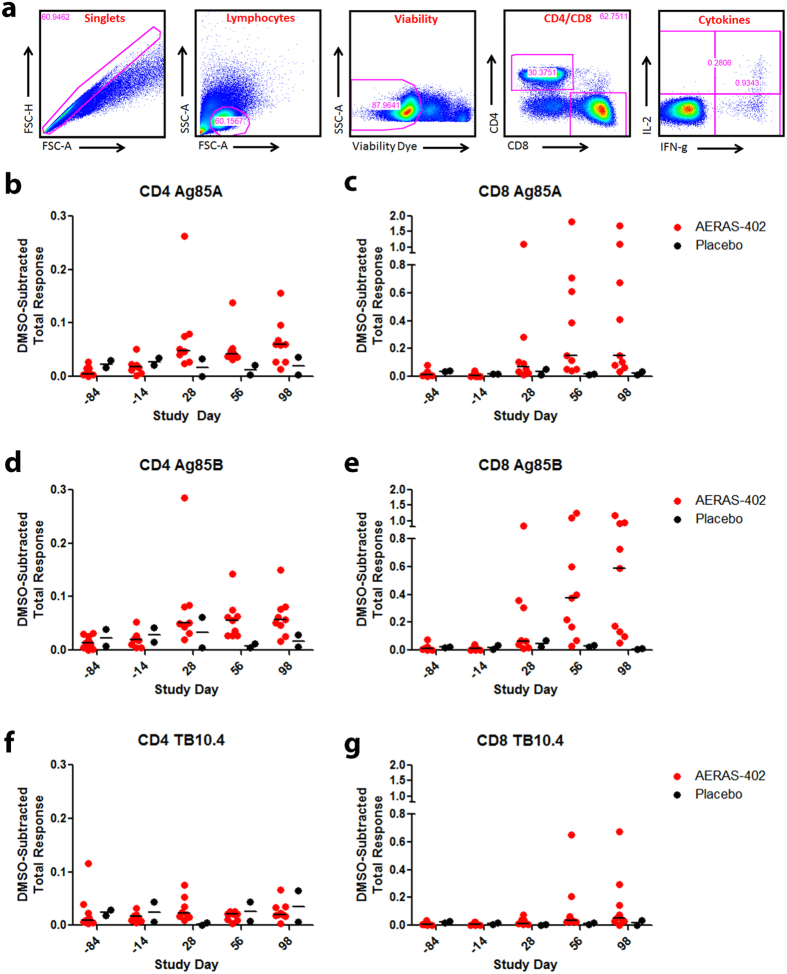
Intracellular Cytokine Staining Response to the AERAS-402 Vaccine. PBMC and leukapheresis specimens from Study Days −84, −14, 28, 56, and 98 were thawed, rested overnight, and stimulated for 5–7 hours with DMSO (negative control), SEB (positive control), or peptide pools corresponding to the vaccine antigens Ag85A, Ag85B, or TB10.4. Specimens were then stained for viability, phenotypic markers, and intracellular cytokine expression and evaluated by flow cytometry. The gating strategy is shown (**a**). The total DMSO-subtracted cytokine response for CD4^+^ and CD8^+^ T cells following stimulation with Ag85A (**b,c**), Ag85B (**d,e**), and TB10.4 (**f,g**). Each circle represents the response from a single participant. Bars represent the median response for each group. Data is shown for participants immunized with BCG on Study Day −84 and placebo (black circles) and participants vaccinated with BCG on Study Day −84 followed by vaccination with AERAS-402 on Study Days 0 and 28 (3 × 10^10^ vp; red circles).

To determine the magnitude and phenotype of vaccine induced immune responses, PBMC, CD8 IFN-γ ELISPOT and ICS was performed using peptide pools (15aa, 11aa overlap) representing each of the three antigens contained within the AERAS-402 vaccine. Most AERAS-402 recipients developed detectable antigen-specific PBMC and CD8 responses by IFN-γ ELISPOT that were further amplified following the second vaccination (Fig. 1). Specifically, the difference for the *ex vivo* PBMC ELISPOT response from day -84 to day 56, reported as the mean, standard error of the mean (SEM), median and IQR (25 to 75 percentile) respectively are [ (Ag85A): 597.1; 378.4; 50.0; 16 to 1315 ], [ (Ag85B): 540.7; 286.6; 60.0; 16 to 1312 ], [ (TB10.4): 243.1; 87.2; 164.0; 13 to 391], compared with the two participants receiving BCG/placebo [ (Ag85A: 55.0; 53.0; 55.0; 2.0 to 108.0], [ (Ag85B): 26.0; 24.0; 26.0; 2.0 to 50.0 ], [ (TB10.4): 47.0; 39.0; 47.0; 8.0 to 86.0 ]. The difference for the *ex vivo* CD8 ELISPOT from day – 84 to day 56, reported as the mean, SEM, median and IQR respectively are [ (Ag85A): 642.7; 290.7; 248.0; 27 to 1177 ], [ (Ag85B): 580.7; 254.0; 228.0; 47 to 1238 ], [ (TB10.4): 245.8; 107.0; 100.0; 16 to 502 ] compared with the two participants receiving BCG/placebo [ (Ag85A): 174.0; 208.0; 174.0; -34 to 382 ], [ (Ag85B): 86.0; 80.0; 86.0; 6 to 166 ], [ (TB10.4): 103.0; 109.0; 103.0; -6 to 212 ]. Although we observe variability in the magnitude of the T cell responses, our findings are similar to the distribution of responses seen in other prime-boost studies^11^,^28^. The PBMC median fold increase for the total cytokine response comparing day -84 to day 98 was 12.5, 3.2, and 1.2 for Ag85A, Ag85B and TB10.4 respectively. The CD8 median fold increase for the total cytokine response comparing day -84 to day 98 was 10.3, 53.4, and 5.6 for Ag85A, Ag85B and TB10.4 respectively (Fig. 2). By comparison, the placebo participants (n = 2) for both CD4^+^ and CD8^+^ T cells had a <1 or negative median fold change (data not shown). Further, these participants generated a polyfunctional cytokine response (Supplementary Fig. S2) consistent with prior immunogenicity studies of AERAS-402^21^,^23^,^24^. We note that BCG vaccination resulted in minimal induction of antigen-specific CD8 responses (Figs 1 and 2) as previously reported^29^.

While most participants had a predictable increase in ELISPOT and cytokine profiles, some participants had a more varied response. Participant 02107 showed a limited response to all of the vaccine antigens while another participant (01805) responded to Ag85A prior to vaccination with BCG. Interestingly, this subject had a more prolonged BCG reaction, suggesting previous exposure to environmental mycobacteria. We also observed a transient, but not insignificant ELISPOT response to Ag85A in a placebo participant at day -14 (00402), which was not observed in the other placebo participant.

### T Cell Clones Generated from AERAS-402 vaccinated participants often do not respond to Mtb-infected cells

**Figure 3:**
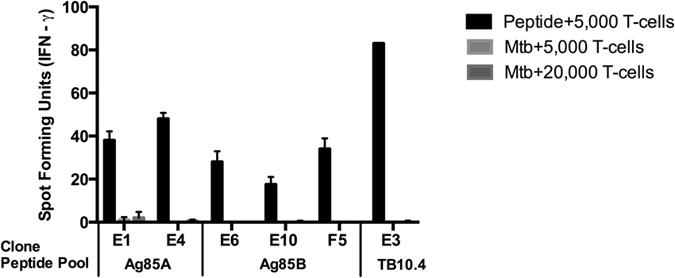
T Cell Clones Respond to Peptide But Not Mtb-infected DC. IFN-γ ELISPOT was performed on 5,000 or 20,000 T cell clones and peptide-pulsed DC (final concentration 5 μg/ml for the respective peptide pools) or Mtb-infected DC (MOI = 30:1) for participant 00201. Error bars represent the standard deviation of the mean of duplicate determinations.

To determine whether or not the CD8^+^ T cells elicited by AERAS-402 were capable of recognizing an Mtb-infected cell, limiting dilution analysis (LDA), using the peptide pools representing the AERAS-402 antigens, and autologous monocyte-derived APC was performed to obtain antigen-specific T cell clones for their ability to recognize either peptide-pulsed or Mtb-infected dendritic cells (DC). Surprisingly, the T cell clones from the initial five participants revealed exclusive reactivity to peptide-pulsed DC and not to Mtb-infected DC. While we have routinely used 5,000 Mtb-infected DC to elicit robust classically and non-classically-restricted Mtb-specific CD8^+^ responses^30^, clones were tested with 20,000 DC per well to ensure adequate availability of Mtb-derived epitopes. Participant 00201 is shown as a representative example (Fig. 3) and none of the T cell clones that responded to peptide responded to the Mtb-infected DC.

Given these unexpected results, we questioned if the epitope recognized by clones derived from peptide pool stimulation might not reflect the full repertoire of epitopes induced in the setting of the AERAS-402 vaccine, and hence might fail to elicit clones capable of recognizing the Mtb-infected cell. Here, we sought to define the breadth of the epitopes elicited by the vaccine. To do this, we performed direct *ex vivo* IFN-γ ELISPOT analysis to define each participant’s CD8^+^ T cell response to each of the individual 15mer peptides that comprised the AERAS-402 peptide pool (Supplementary Fig. S3). Here, we show that participants vaccinated with AERAS-402 have distinct but focused reactivity to each of the 15mer peptides (Supplementary Fig. S3). Where sufficient PBMC were available, we sought to generate an expanded panel of T cell clones that would better reflect the breadth of these responses. We then selected individual 15mer peptides that elicited a robust *ex-vivo* response to generate T cell clones. In this case, either traditional LDA cloning^31^ or a novel CFSE-based method was employed and 19 stable CD8^+^ T cell clones were derived (Table 4 and illustrated by+ in Supplementary Fig. S3 for the cognate 15mer). In one instance, (participant 01304, clone B12; Ag85A_133–147_) the epitope did not correlate with the *ex-vivo* analysis likely the result of cross reactivity with peptide for Ag85B_133–147_. Each clone was tested for its ability to recognize Mtb-infected cells and, where possible, the restricting allele and minimal epitope recognized was defined (Supplementary Fig. S4).

**Figure 4:**
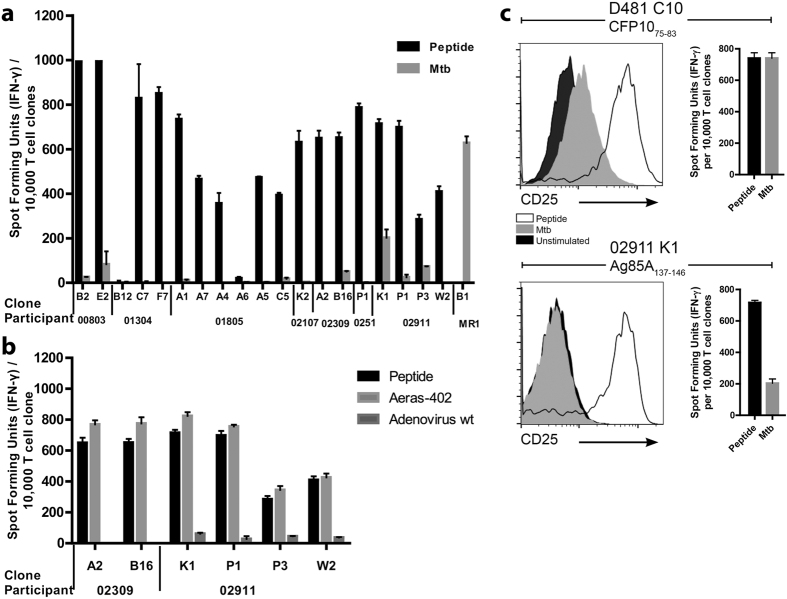
T cell clones generated from AERAS-402 vaccinated donors do respond to AERAS-402 vaccine, but do not respond to Mtb-infected DC. 10,000 T cell clones were incubated in the presence of 0.5 ng/ml IL-2 overnight in an ELISPOT with (**a**) autologous DC pulsed with 5 ug/ml peptide or DC infected (MOI 30:1) with Mtb. The participant labeled MR1 refers to the MR-restricted clone B1 that recognizes Mtb-infected target and is the positive control. To assess if clones weakly or non-responsive to the Mtb-infected target would also be less responsive to an adenovirally-infected target, 10,000 T cell clones were incubated in the presence of 0.5 ng/ml IL-2 overnight in an ELISPOT with autologous DC pulsed with 5 ug/ml peptide, DC infected with adenovirus containing AERAS-402 and adenovirus wildtype (**b**) and tested for their ability to elicit IFN-γ. The respective peptide for each T cell clone is defined in Table 4. Error bars represent the standard deviation of the mean of duplicate determinations. (**c**) 100,000 T cell clones were incubated overnight with autologous DC pulsed with 5 ug/ml peptide or DC infected (MOI 30:1) with Mtb for 24 hours and then stained with CD3, CD8, CD4, Live/Dead discriminator and CD25 and then gated on Live, CD3^+^, CD4^−^, CD8^+^ cells.

While all clones recognized their cognate peptide, three distinct patterns of Mtb recognition were observed (Fig. 4a). First, we observed clones incapable of recognizing the Mtb-infected target (n = 14 (74%)). Second, were clones with a low Mtb-specific response relative to peptide (E2 (00803), B16 (02309), P1 and P3 (02911). Third, one clone had a robust response to Mtb when compared to other clones (K1 for participant 02911), albeit still less than the peptide response. For comparison, the MR1-restricted (HLA-Ia unrestricted) clone B1 has been included as a positive control as it is known to respond to the Mtb-infected cells from all of the donors enrolled.

### Mtb Unresponsive Clones Efficiently Recognize Adenovirally Presented Antigen

We reasoned that those clones weakly or non-responsive to the Mtb-infected target might be those with a low-affinity T cell receptor, which in turn would exhibit diminished responsiveness to an adenovirally-infected target. To test this, DC were infected with either the AERAS-402 adenovirus or the wild type adenovirus control, and tested for their ability to elicit IFN-γ. All of the clones were strongly responsive to the AERAS-402 vaccine, and we observed no relationship between the degree of responsiveness and the response to Mtb-infected targets (Fig. 4b). While each clone was screened based on the production of IFN-γ, we sought to address the concern that the Mtb-infected target could induce T cell activation, but not IFN-γ release. To test this, 100,000 T cell clones were incubated overnight with autologous DC, and assessed for markers associated with T cell activation. Specifically, DC were pulsed with 5 ug/ml peptide or DC infected (MOI 30:1) with Mtb for 24 hours and then stained with CD3, CD8, CD4, Live/Dead discriminator and the activation marker CD25. In comparison to a clone generated using an Mtb infected DC (D481 C10; CFP10_75–83_)^15^, we observed that stimulation of the AERAS-402 clone (02911 K1 Ag85_137–146_) with the Mtb-infected DC resulted in minimal upregulation of CD25 (Fig. 4c). These data are concordant with the IFN-γ data shown, in which the CFP-specific clone elicited comparable IFN-γ release in response to either peptide-pulsed or Mtb-infected DC, while the Ag85 specific clone preferentially released IFN-γ in response to the peptide-pulsed target. While we did not specifically measure other cytokines or components of the granule exocytosis pathway, we note that T cell activation is a prerequisite for the release of these markers.

### Preconditioning antigen presenting cells with proinflammatory cytokines TNF-α or IFN-γ does not increase recognition of Mtb-infected cells

We next tested the hypothesis that induction of the immune-proteasome^32^ might be required for the processing and presentation of Mtb-derived epitopes. Neither TNF-α nor IFN-γ preconditioning of the Mtb-infected DC affected recognition (Supplementary Fig. S5). Finally we postulated that T cell cloning led to the generation of low-affinity T cells. To test this hypothesis, an identical protocol to that described was used to generate a TB10.4 specific T cell clone (aa 73–87; SSTHEANTMAMMARD) from a subject with latent TB infection. The resulting T cell clone responded similarly to its cognate epitope and to the Mtb-infected DC (Supplementary Fig. S6).

## Discussion

While the AERAS-402 vaccine after BCG prime is broadly immunogenic^21^,^23^,^24^, the observation that vaccine-elicited CD8^+^ T cells were in many instances not capable of recognizing an Mtb-infected cell raises the possibility that enumeration of vaccine-induced polyfunctional T cells may not reflect the extent or degree to which these cells are capable of identifying cells infected with Mtb. At present, correlates of protective immunity for Mtb are poorly understood, and yet desperately needed to guide improved vaccine design. Our data would support supplemental approaches to define vaccine-induced immunity for Mtb^3^,^33^. In addition to the evaluation of vaccine-induced T cells to recognize the infected cells, these approaches might include direct measurement of Mtb growth inhibition proposed as an adjunct to immunogenicity studies^34^,^35^. Similarly, our findings would also support the use of leukapheresis in conjunction with Phase I vaccination studies as a platform for comprehensive analysis of vaccine-induced immunity. In this regard, detailed experimental medicine studies, early in vaccine development would allow for a more rational and robust approach in the definition of surrogates of protective immunity in the context of larger Phase IIb studies, and ultimately the prioritization of novel vaccine candidates.

Collective evidence reinforces the fundamental biologic capacity of the CD8^+^ T cell response to preferentially discern high burden intracellular infection with Mtb^14^,^36^. However, this study does not identify the specific mechanisms underlying the reasons that some of the vaccine-elicited CD8^+^ T cells did not recognize epitopes displayed by the Mtb-infected cell. One possibility is that antigens that are presented by the AERAS-402 vaccine have limited access to the HLA-Ia processing machinery during the course of Mtb infection *in vivo*. This could be the result of protein abundance, tissue distribution, or access to the HLA-Ia processing machinery. In this regard, while many antigens elicit high-frequency T cell responses in the setting of natural infection^37^ we have observed previously that Ag85, one of the antigens in AERAS-402, is a relatively poor inducer of CD8^+^ T cells^15^. Similarly, Lindestrom *et al*. report the structure of TB10.4 limits effective proteosomal and epitope processing^38^. In any case, the determination and subsequent use of antigens associated with robust Mtb-specific CD8^+^ T cell responses might improve the ability of virally delivered vaccines to elicit epitopes displayed during the course of infection with Mtb.

Alternately, the immunodominance hierarchy elicited by adenoviral vaccination may not reflect that elicited in the course of natural infection. In this regard, the relatively high abundance of cytosolic proteins found during adenoviral infection may allow for the selection of epitopes that may be rare or less relevant^39^ during natural infection with Mtb. Mice vaccinated with an Ag85B-ESAT-6 fusion molecule delivered via an adenoviral vector had a strong IFN-γ mediated recall response to vaccine-induced epitopes however, this response was ultimately non- protective. Strikingly, modifying the viral delivery system impacted the immune dominance hierarchy toward a more protective phenotype^39^. Thus, the immunodominance hierarchy presented in Supplementary Fig. S3 may not reflect a profile that may be seen in the same individual following natural infection with Mtb. Similarly, this study was conducted in subjects without a history of BCG vaccination prior to enrollment, thus we cannot ascertain if remote BCG^21^,^25^ prior to an adenoviral boost would have changed the immunodominance hierarchy seen herein. Specifically, it is possible that those epitopes elicited during the course of natural infection with BCG would be preferentially expanded by the subsequent adenoviral challenge. Counter to this argument, is the observation by ourselves and others that BCG is a poor inducer of Ag85-specific CD8^+^ T cell responses.

Additionally, a limitation of this study is that sub dominant or “cryptic” epitopes might nonetheless be augmented and hence protective following natural infection with Mtb, as shown in the murine model^40^,^41^,^42^. In the approach taken in this report, we have likely failed to map those sub dominant epitopes. Here we note that an epitope that might be considered immune dominant for one individual was often sub dominant for another, precluding a broader classification of immunodominance. Further, we may not have accounted for the full repertoire of T cell effector function. Specifically, we have based our original screen on the production of IFN-γ, but did not more directly assess for GM-CSF, IL-1β, granulysin, granzyme B or perforin. In this regard, we note that evaluation of markers of T cell activation (Fig. 4) directly *ex-vivo* would provide a means to assess the ability of vaccine induced CD8^+^ T cells to recognize the Mtb-infected cell.

In summary, while AERAS-402 is broadly immunogenic, our findings highlight that measurement of vaccine-induced, polyfunctional T cells may not reflect the extent or degree to which these cells are capable of identifying the Mtb-infected cell. Detailed experimental medicine studies paired with new vaccine studies may accelerate a more complete understanding of patterns of immune dominance^43^ and subdominance and thus inform correlates of protective immunity induced by vaccination.

## Methods

### Protocol

A Phase I, Double Blind, Randomized, Placebo-controlled Leukapheresis Study to Obtain Lymphocytes for the Study of Immune Responses in Healthy Adult Volunteers in the U.S. who receive BCG Vaccination followed by Boosting with AERAS-402.

Investigational Product: AERAS-402

Aeras Protocol Number: C-021-402

US FDA IND Number: BB-IND 13140

### Experimental Design

We performed a Phase I, double-blind, randomized, placebo-controlled study in a group of healthy adult male and female participants who are HIV-negative, BCG-unvaccinated and have no evidence of tuberculosis infection.

### Registration number and Trial Registry Information

The study was registered with Clinicaltrials.gov and has been given the identifier of NCT02375256. Clinicaltrials.gov is an approved registry by the International Committee of Medical Journal Editors (ICMJE), which is one of the platforms listed by the World Health Organization International Clinical Trials Registry Platform (ICTRP). The study was retrospectively added to ClinicalTrials.gov on 2/24/15. At study initiation, the trial did not meet the requirements for an “applicable clinical trial” under the FDAAA 801 thus did not need to be added at the time. The trial was retrospectively registered to meet the publication requirements of ICMJE.

### Primary Objective(s)

The primary objective of this study is to evaluate the cellular immune responses to AERAS-402 in healthy adult volunteers who receive two booster doses of AERAS-402 administered 84 and 112 days after BCG vaccination, through leukapheresis and cryopreservation of cells followed by *in vitro* assays.

### Participants

Participants were eligible for enrollment if they provided informed consent prior to any study related procedures. We enrolled healthy adults aged 18–45, who had BMI of ≥19 and <33, who were not pregnant and agreed to avoid pregnancy during the study period. Participants were excluded if they had signs and symptoms of acute illness including a temperature over 37.5 °C or lymphadenopathy, abnormal hemoglobin or hematocrit, abnormal white blood cell count, absolute neutrophil count, or absolute lymphocyte count, elevated creatinine, total bilirubin, AST, ALT, or alkaline phosphatase at Study Day -84, who had evidence of chronic hepatitis, who had evidence of active or latent TB (QuantiFERON-TB (QFT), X-ray), who resided in an endemic TB area or with a person with confirmed TB, who has a history of alcohol or drug abuse and who in the opinion of the investigator had any medical history that may compromise the safety of the subject in the study. Demographic characteristics of participants enrolled as well as reasons for exclusion are outlined in Tables 1 and 2.

### Study setting, Participant Consent and Follow Up

The study was performed at Oregon Health and Science University (OHSU) in Portland, Oregon, USA. The study was approved by the OHSU Institutional Review Board. Written informed consent was obtained from each subject prior to the conduct of any protocol specific activity or study entry. Before providing such consent, each subject was informed of the nature and purpose of the study, potential risks and benefits of study participation, the procedures to be performed as part of the study, and of their right to withdraw from the study at any time without risk of retribution. The study was conducted according to the Declaration of Helsinki, ICH-GCP, Protection of Human Subjects (21 CFR 50), Institutional Review Boards (21 CFR 56), Obligations of Clinical Investigators (21 CFR 312), and local regulatory requirements.

The total duration of follow up after randomization was 98 days for each subject. The first BCG vaccination was on 28/Oct/2009. Subjects were randomized to placebo or AERAS-402 between 21/Jan/2010 and 01/Sep/2010. The final follow up visit was on 01/Dec/2010.

### Sample Size, Randomization and Blinding

The sample size for this study was selected as adequate for initial assessment of the utility of leukapheresis rather than for statistical reasons and allowed for a preliminary review of cellular immune responses following vaccination with AERAS-402. Participants were randomized to a treatment assignment using a paper based randomization schedule created by a statistician not involved with the analysis of the study in order to maintain blinding of the study team. Participants were randomized 10:3 to AERAS-402 or placebo (sterile vaccine buffer) on Study Day 0. Doses were prepared by an un-blinded pharmacist. Participants and all site and laboratory personnel were blinded to subject assignment. Un-blinding did not occur until study completion and after completion of all immunology studies.

### Summary Schedule and Safety Evaluations

Thirteen eligible participants were vaccinated with 1–8 × 10^5^ cfu of BCG intradermally at Study Day -84. On Study Day 0, eleven eligible participants were randomized to receive either AERAS- 402 at 3 × 10^10^ vp or placebo intramuscularly into the deltoid on the opposite arm of the BCG vaccination. Participants received the same study vaccine (AERAS-402 or Placebo) on Study Day 28. Participants were followed for adverse events for 28 days following each vaccination of AERAS- 402 or placebo and for SAEs for the entire study period. Peripheral blood for routine hematology and chemistries and urine for urinalysis were taken from each participant prior to each vaccination (Study Days 0, 28), seven days after each vaccination (Study Days 7, 35) and at Study Day 56. Peripheral blood was collected for immunogenicity by ICS/ELISPOT on Study Days -84, -14, 28 and 56. Blood was also collected on day 98 for ICS. Leukapheresis samples were collected at study days -14 and between days 56 and 98. QuantiFERON (QFT^®^) and HIV testing were performed at screening and again at the completion of the study. A full schedule of participant evaluations is shown in Supplementary Table S1.

### Immunology Studies

Leukapheresis product was washed three times with PBS with 1 mM EDTA, processed using histopaque (Sigma), and the buffy coat collected and washed three times with PBS and frozen.

#### Generation and infection of peripheral blood DC and Macrophages

Monocyte-derived DC were prepared according to a modified method of Romani *et al*.^44^ Briefly, PBMC obtained by apheresis were resuspended in 2% human serum (HS) in RPMI and allowed to adhere to a T-75 (Costar) flask at 37 °C for 1 h. After gentle rocking, nonadherent cells were removed and 10% HS in RPMI containing 10 ng/ml of IL-4 (R&D Systems) and 30 ng/ml of GM-CSF (Immunex) was added to the adherent cells. After 5 days, cells were harvested with cell dissociation medium (Sigma-Aldrich) and used as antigen presenting cells (APC) in assays. Macrophages were generated by purifying CD14^+^ cells from PBMC using a CD14 positive selection kit (Stemcell Technologies) and incubated for 5 days and used as APC in assays. To generate Mtb-infected DC, cells (1 × 10^6^) were cultured overnight in the presence of Mtb (strain H37Rv) at a multiplicity of infection of 30:1. We have determined that this multiplicity of infection is optimal for detection of Mtb-specific CD8^+^ T cells, as heavy infection is required to optimize entry of antigen into the class I processing pathway^14^. After 18 hours, the cells were harvested and resuspended in 10% HS in RPMI.

#### IFN-γ ELISPOT assay

The IFN-γ ELISPOT assay was performed as described previously^27^. *Ex vivo* frequencies of CD8^+^ T cells responding to Mtb antigens were determined using two methods. First, CD4^+^ T cells were depleted using magnetic beads (Stemcell Technologies) and 250,000 CD4-depleted PBMC per well were tested in duplicate and incubated with peptide pool (5 μg/ml) overnight (CD8/others). Second, CD8^+^ T cells were positively selected from PBMC using magnetic beads (Stemcell Technologies) such that >97% of the cell population were CD8^+^ T cells. These CD8^+^ T cells were used as a source of responder T cells and tested in duplicate at a cell concentration of 250,000 cells per well. Autologous DC (20,000 cells/well) were used as APC and peptide pools (5 μg/ml, final concentration of each peptide) were added to the assay (CD8/DC). For assays using T cell clones, T cells (1,000, 5,000, or 10,000 cells/well) were incubated with autologous LCL or DC (20,000 cells/well) in the presence or absence of antigen or incubated with DC infected with Mtb (MOI 30:1). Negative and positive controls were included in all assays and consisted of wells containing T cells and DC either without antigen or without antigen but with inclusion of phytohemagglutanin (PHA, 10 μg/ml), respectively. For all assays, IFN-γ was assessed by ELISPOT after 18 hours of co-culture.

#### ICS Studies

ICS studies were performed as described previously^24^. PBMC and leukapheresis specimens from Study Days -84, -14, 28, 56, and 98 were thawed, rested overnight, and stimulated for 5–7 hours with DMSO (negative control), SEB (positive control), or peptide pools corresponding to the vaccine antigens Ag85A, Ag85B, or TB10.4. Specimens were then stained for viability, phenotypic markers, and intracellular cytokine expression and evaluated by flow cytometry. The total DMSO-subtracted cytokine response for CD4^+^ and CD8^+^ T cells following stimulation with Ag85A, Ag85B, and TB10.4 were calculated. Boolean gates were generated for each of the stimulation conditions followed by DMSO subtraction. Total responses were then calculated by summing the DMSO-subtracted gates.

#### Cloning Methods

Peptide-specific T cell clones were isolated using peptide-pulsed DC as APC and limiting dilution cloning methodology as previously described^15^. Briefly, CD8^+^ T cells were isolated from PBMCs using positive selection with CD8 antibody-coated magnetic beads per the manufacturer’s instructions (Miltenyi Biotec http://www.miltenyibiotec. com). T cells were seeded at various concentrations in the presence of a 1 × 10^5^ irradiated autologous peptide pool-pulsed DC, generated as described above, and rIL-2 (5 ng/ml) in cell culture media consisting of 200 μl of RPMI 1640 supplemented with 10% human sera. Wells exhibiting growth between 10–14 days were assessed for peptide pool specificity using ELISPOT and peptide-pool pulsed DC as a source of APC. T cells retaining peptide pool specificity were expanded using our standard rapid expansion protocol as described below. After expansion, these T cell clones were tested for reactivity to the peptide pool and Mtb-infected DC by ELISPOT.

CFSE (carboxyfluorescein succinimidyl ester) T cell cloning was performed as follows. First, having identified the 15-mer(s) of interest, a CD8^+^ T cell line was created by incubating 1 × 10^6^ CD8^+^ T cells with 1 × 10^5^ peptide-pulsed DC per well of a 24 well plate. On day 7, this line was collected, stained with CFSE (5-(and 6)-Carboxyfluorescein diacetate succinimidyl ester) and added to a 24 well plate at a concentration of 1 × 10^6^ per well and re-stimulated with 1 × 10^5^ peptide-pulsed macrophages, generated as described above. On day 12, the T cell line was sorted for cells that had divided in response to the peptide-pulsed macrophages and are therefore CFSE dilute. Between one and five million CFSE dilute, CD8^+^ T cells were collected and rested for 2–3 days with rIL-2 (0.5 ng/ml). These are now candidate T cell clones as all of the T cells expanded in response to a single 15mer peptide. These T cell clones were assessed for peptide specificity and Mtb-reactivity using ELISPOT and peptide-pulsed or Mtb-infected DC as a source of APC.

Some CD8^+^ T cell lines were generated using peptide pools instead of an individual 15mer peptide. A CD8^+^ T cell line was created as described above, but CFSE was not added on day 7. For these T cell lines, we performed a modified LDA. T cells were seeded at various concentrations in the presence of a 1 × 10^5^ irradiated PBMC, 3 × 10^4^ irradiated LCL, rIL-2 (5 ng/ml) and anti-CD3 mAb (30 ng/ml) in cell culture media consisting of 200 μl of RPMI 1640 supplemented with 10% human sera. Wells exhibiting growth between 10–14 days were assessed for peptide pool specificity using ELISPOT and peptide-pool pulsed DC as a source of APC. T cells retaining peptide pool specificity were expanded using our standard rapid expansion protocol described below. After expansion, these T cell clones were tested for reactivity to the peptide pool and Mtb-infected DC by ELISPOT.

#### Expansion of T Cell Clones

To expand the CD8^+^ T cell clones, a rapid expansion protocol using anti-CD3 mAb stimulation was used as previously described^27^. Briefly, T cell clones were cultured in the presence of irradiated allogeneic PBMCs (25 × 10^6^), irradiated allogeneic LCL (5 × 10^6^), and anti-CD3 mAb (30 ng/ml; Orthoclone OKT3) in RPMI 1640 media with 10% human serum in a T-25 upright flask in a total volume of 30 ml. The cultures were supplemented with IL-2 (1 ng/ml; Proleukin, Prometheus) on days 1, 4, 7, and 10 of culture. The cell cultures were washed on day 5 to remove remaining soluble anti-CD3 mAb. All expanded T-cell clones are routinely tested for mycoplasma infection.

### Analyses

Immune cells from peripheral blood and leukapheresis product were analyzed for the presence and functional capacity of antigen-specific cells by antigen stimulation followed by visualization and quantitation of cytokine –producing cells using enzyme-linked immunospot (ELISPOT) techniques and ICS analysis. Responses are summarized using descriptive statistics.

For ICS, the variables of interest for assessment of immune response to AERAS-402 are the percentage of CD4^+^ and CD8^+^ T cells from leukapheresis that produce any of three cytokines (IFN-γ, TNF-α, and/or IL-2) (Supplementary Fig. S2) or a combination of the three cytokines simultaneously following stimulation with peptide pools (Fig. 2) derived from and representing the entire amino acid sequences of the mycobacterial antigens Ag85A and parts of Ag85B (Ag85A/b), and the complete pool of TB10.4. Responses are measured by flow cytometry in the intracellular cytokine staining (ICS) assay. Summaries include immune response at all available pre- and post-vaccination immunology time points. Data were analyzed using FlowJo software to generate cytokine Boolean gates. Each gate was subjected to DMSO subtraction to remove background and plotted as the total ICS response (Fig. 2) or the percent response (Supplementary Fig. 2) of CD4^+^ or CD8^+^ T cell populations.

IFN-γ ELISPOT analysis is shown as spot forming units per 250,000 T cells unless otherwise specified in figure legends. All determinations were performed in duplicate and plotted values represent the mean of the respective determinations less background. Error bars represent the standard deviation of duplicate determinations.

The analysis provided is exploratory and descriptive. Descriptive statistics (mean, standard error of the mean (SEM), median and interquartile range (25^th^ to 75^th^ percentile) and/or count (percentage) summaries are used to summarize the results. All results are summarized by vaccination regimen.

## Additional Information

**How to cite this article**: Nyendak, M. *et al*. Adenovirally-Induced Polyfunctional T Cells Do Not Necessarily Recognize the Infected Target: Lessons from a Phase I Trial of the AERAS-402 Vaccine. *Sci. Rep.* **6**, 36355; doi: 10.1038/srep36355 (2016).

**Publisher’s note:** Springer Nature remains neutral with regard to jurisdictional claims in published maps and institutional affiliations.

## Supplementary Material

Supplementary Information

## Figures and Tables

**Table 1:** Summary of patient baseline characteristics (*n = 11).*

Characteristics	Statistics, n (%)¶
Gender	
Female	5 (45.5)
Male	6 (54.5)
Age, median	37.0
Race	
American Indian or Alaska Native	1 (9.1)
Asian	1 (9.1)
Black or African American	0 (0.0)
White	9 (81.8)
Ethnicity	
Hispanic or Latino	1 (9.1)
Not Hispanic or Latino	10 (90.9)
BMI, mean (standard deviation)	24.28 (3.85)

^¶^Total participants enrolled: 11 (100%), 9 receiving AERAS-402 and 2 placebo.

**Table 2:** Reasons for Exclusion of Screened Participants.

Reason for Screening Failure	*n* (%)¶
Laboratory Abnormalities	6 (33.33)
Lack of general good health as confirmed by medical history and physical exam	5 (27.78)
High risk sexual history	2 (11.11)
Declined to participate	1 (6.56)
BMI	1 (6.56)
Cannabis use	1 (6.56)
Positive QFT	1 (6.56)
Concurrent enrollment in another investigational study	1 (6.56)

^¶^Total individuals screened: 29. Total Screen Failures: 18 (62% of screened participants).

**Table 3:** Short Summary of Participant Vaccination and Immunology Evaluations.

Evaluation	Screen (45 Days)	Study Day											
		−84	−77	−56	−14	0	7	14	28	35	42	56	98
**BCG administration**		**x**											
**AERAS-402/placebo administration**						**x**			**x**				
Immunology: ICS^b^, ELISPOT		x			x				x			x	
Leukapheresis					x								x^a^

^a^Study Day 98 leukapheresis was done any time after the Study Day 56 visit but no later than the Study Day 98 visit. Note: The amount of blood required for this study is within WHO guidelines for blood donation.

^b^ICS was also performed on day 98.

**Table 4:** Summary of T Cell Clones.

Participant	Clone^A^	Protein	HLA-Restricting Allele	Epitope Location	Epitope Sequence^B^	Epitope Specific T cells^C^
00803	B2 (0)	Ag85A	B2705	185–203	DPAWQRNDPLLNVGKLIAN	nd
00803	E2 (2)	Ag85B	A2402	97–111	WETFLTSELPQWLSA	nd
01304	B12 (0)	Ag85A	nd	133–147	LTLAIYHPQQFVYAG	nd
01304	C7 (0)	Ag85A	nd	253–267	VFDFPDSGTHSWEYW	nd
01304	F7 (0)	Ag85A	nd	249–267	GHNGVFDFPDSGTHSWEYW	nd
01805	A1 (0)	Ag85A	nd	69–83	VMPVGGQSSFYSDWY	nd
01805	A7-1 (5)	Ag85A	B3501	65–83	GLSVVMPVGGQSSFYSDWY	nd
01805	A4-2 (4)	Ag85B	nd	65–79	GLSIVMPVGGQSSFY	nd
01805	A6-2 (0)	Ag85B	nd	249–263	GHNAVFNFPPNGTHS	nd
01805	A5-3 (0)	TB10.4	nd	57–71	QWNQAMEDLVRAYHA	nd
01805	C5-3 (0)	TB10.4	nd	37–59	AALQSAWQGDTGITYQAWQAQWN	nd
02107^D^	K2 (0)	Ag85A	B0702	116–123	KPTGSAVV	64
02309	A2 (0)	Ag85A	B5601	6–14	LPVEYLQVP	81
02309	B16 (0)	Ag85B	C1202	62–70	YQSGLSIVM	251
02510^D^	P1 (1)	Ag85B	A0206	62–70	YQSGLSIVM	442
02911	K1 (0)	Ag85A	C0702	137–146	IYHPQQFVYA	244
02911	P1 (1)	Ag85B	B3901	62–70	YQSGLSIVM	343
02911	P3 (0)	Ag85B	C0702	137–145	AYHPQQFIY	200
02911	W2 (0)	TB10.4	B3901	75–83	THEANTMAM	147

^A^Number of sister clones is in parentheses.

^B^The minimal epitope is yet to be determined.

^C^IFN-γ spot forming units per 250,000 CD8^+^ T cells. nd: not done.

^D^MHC binding affinity (IC50 nm) for 02107 = 10 and for 02510 = 45.

## References

[b1] Dye C., Glaziou P., Floyd K. & Raviglione M. Prospects for tuberculosis elimination. Annu Rev Public Health 34, 271–286, doi: 10.1146/annurev-publhealth-031912-114431 (2013). 23244049

[b2] Ernst J. D. The immunological life cycle of tuberculosis. Nat Rev Immunol 12, 581–591, doi: 10.1038/nri3259 (2012). 22790178

[b3] Nunes-Alves C. . In search of a new paradigm for protective immunity to TB. Nat Rev Microbiol 12, 289–299, doi: 10.1038/nrmicro3230 (2014). 24590243 PMC4085047

[b4] Cooper A. M. Cell-mediated immune responses in tuberculosis. Annu Rev Immunol 27, 393–422, doi: 10.1146/annurev.immunol.021908.132703 (2009). 19302046 PMC4298253

[b5] O’Garra A. . The immune response in tuberculosis. Annu Rev Immunol 31, 475–527, doi: 10.1146/annurev-immunol-032712-095939 (2013). 23516984

[b6] Hokey D. A. & Ginsberg A. The current state of tuberculosis vaccines. Hum Vaccin Immunother 9, 2142–2146, doi: 10.4161/hv.25427 (2013). 23792698 PMC3906398

[b7] Lindenstrom T. . Tuberculosis subunit vaccination provides long-term protective immunity characterized by multifunctional CD4 memory T cells. J Immunol 182, 8047–8055, doi: 10.4049/jimmunol.0801592 (2009). 19494330

[b8] Beveridge N. E. . Immunisation with BCG and recombinant MVA85A induces long-lasting, polyfunctional Mycobacterium tuberculosis-specific CD4+ memory T lymphocyte populations. Eur J Immunol 37, 3089–3100, doi: 10.1002/eji.200737504 (2007). 17948267 PMC2365909

[b9] Wilkinson K. A. & Wilkinson R. J. Polyfunctional T cells in human tuberculosis. Eur J Immunol 40, 2139–2142, doi: 10.1002/eji.201040731 (2010). 20853500

[b10] Seder R. A., Darrah P. A. & Roederer M. T-cell quality in memory and protection: implications for vaccine design. Nat Rev Immunol 8, 247–258, doi: 10.1038/nri2274 (2008). 18323851

[b11] Tameris M. D. . Safety and efficacy of MVA85A, a new tuberculosis vaccine, in infants previously vaccinated with BCG: a randomised, placebo-controlled phase 2b trial. Lancet 381, 1021–1028, doi: 10.1016/S0140-6736(13)60177-4 (2013). 23391465 PMC5424647

[b12] Lewinsohn D. A., Gold M. C. & Lewinsohn D. M. Views of immunology: effector T cells. Immunol Rev 240, 25–39, doi: 10.1111/j.1600-065X.2010.00997.x (2011). 21349084

[b13] Lewinsohn D. M. . Human purified protein derivative-specific CD4+ T cells use both CD95-dependent and CD95-independent cytolytic mechanisms. J Immunol 160, 2374–2379 (1998). 9498779

[b14] Lewinsohn D. A. . Mycobacterium tuberculosis-specific CD8+ T cells preferentially recognize heavily infected cells. Am J Respir Crit Care Med 168, 1346–1352, doi: 10.1164/rccm.200306-837OC200306-837OC [pii] (2003). 12969871

[b15] Lewinsohn D. A. . Immunodominant tuberculosis CD8 antigens preferentially restricted by HLA-B. PLoS Pathog 3, 1240–1249, doi: 10.1371/journal.ppat.0030127 (2007). 17892322 PMC2323292

[b16] Cho S. . Antimicrobial activity of MHC class I-restricted CD8+ T cells in human tuberculosis. Proc Natl Acad Sci USA 97, 12210–12215, doi: 10.1073/pnas.210391497 (2000). 11035787 PMC17320

[b17] Flynn J. L., Goldstein M. M., Triebold K. J., Koller B. & Bloom B. R. Major histocompatibility complex class I-restricted T cells are required for resistance to Mycobacterium tuberculosis infection. Proc Natl Acad Sci USA 89, 12013–12017 (1992). 1465432 10.1073/pnas.89.24.12013 PMC50688

[b18] Lalvani A. . Human cytolytic and interferon gamma-secreting CD8+ T lymphocytes specific for Mycobacterium tuberculosis. Proc Natl Acad Sci USA 95, 270–275 (1998). 9419365 10.1073/pnas.95.1.270 PMC18198

[b19] Lewinsohn D. M. . Characterization of human CD8+ T cells reactive with Mycobacterium tuberculosis-infected antigen-presenting cells. J Exp Med 187, 1633–1640 (1998). 9584141 10.1084/jem.187.10.1633 PMC2212289

[b20] Lin P. L. & Flynn J. L. CD8 T cells and Mycobacterium tuberculosis infection. Semin Immunopathol 37, 239–249, doi: 10.1007/s00281-015-0490-8 (2015). 25917388 PMC4439333

[b21] Abel B. . The novel tuberculosis vaccine, AERAS-402, induces robust and polyfunctional CD4+ and CD8+ T cells in adults. Am J Respir Crit Care Med 181, 1407–1417, doi: 10.1164/rccm.200910-1484OC (2010). 20167847 PMC2894413

[b22] Havenga M. . Novel replication-incompetent adenoviral B-group vectors: high vector stability and yield in PER.C6 cells. J Gen Virol 87, 2135–2143, doi: 10.1099/vir.0.81956-0 (2006). 16847108

[b23] Hoft D. F. . A recombinant adenovirus expressing immunodominant TB antigens can significantly enhance BCG-induced human immunity. Vaccine 30, 2098–2108, doi: 10.1016/j.vaccine.2012.01.048 (2012). 22296955

[b24] Kagina B. M. . The novel tuberculosis vaccine, AERAS-402, is safe in healthy infants previously vaccinated with BCG, and induces dose-dependent CD4 and CD8T cell responses. Vaccine 32, 5908–5917, doi: 10.1016/j.vaccine.2014.09.001 (2014). 25218194

[b25] Churchyard G. J. . The safety and immunogenicity of an adenovirus type 35-vectored TB vaccine in HIV-infected, BCG-vaccinated adults with CD4(+) T cell counts >350 cells/mm(3). Vaccine 33, 1890–1896, doi: 10.1016/j.vaccine.2015.02.004 (2015). 25698492

[b26] Schulz K. F., Altman D. G. & Moher D. CONSORT 2010 statement: updated guidelines for reporting parallel group randomized trials. Ann Intern Med 152, 726–732, doi: 10.7326/0003-4819-152-11-201006010-00232 (2010). 20335313

[b27] Heinzel A. S. . HLA-E-dependent presentation of Mtb-derived antigen to human CD8+ T cells. J Exp Med 196, 1473–1481 (2002). 12461082 10.1084/jem.20020609 PMC2194265

[b28] Tameris M. . A double-blind, randomised, placebo-controlled, dose-finding trial of the novel tuberculosis vaccine AERAS-402, an adenovirus-vectored fusion protein, in healthy, BCG-vaccinated infants. Vaccine 33, 2944–2954, doi: 10.1016/j.vaccine.2015.03.070 (2015). 25936724 PMC6698638

[b29] Kagina B. M. . Specific T cell frequency and cytokine expression profile do not correlate with protection against tuberculosis after bacillus Calmette-Guerin vaccination of newborns. Am J Respir Crit Care Med 182, 1073–1079, doi: 10.1164/rccm.201003-0334OC (2010). 20558627 PMC2970848

[b30] Harriff M. J. . Human lung epithelial cells contain Mycobacterium tuberculosis in a late endosomal vacuole and are efficiently recognized by CD8(+) T cells. PLoS One 9, e97515, doi: 10.1371/journal.pone.0097515 (2014). 24828674 PMC4020835

[b31] Gold M. C. . Human mucosal associated invariant T cells detect bacterially infected cells. PLoS Biol 8, e1000407, doi: 10.1371/journal.pbio.1000407 (2010). 20613858 PMC2893946

[b32] Basler M., Kirk C. J. & Groettrup M. The immunoproteasome in antigen processing and other immunological functions. Curr Opin Immunol 25, 74–80, doi: 10.1016/j.coi.2012.11.004 (2013). 23219269

[b33] Kaufmann S. H. Fact and fiction in tuberculosis vaccine research: 10 years later. Lancet Infect Dis 11, 633–640, doi: 10.1016/S1473-3099(11)70146-3 (2011). 21798463

[b34] Hoft D. F. . Investigation of the relationships between immune-mediated inhibition of mycobacterial growth and other potential surrogate markers of protective Mycobacterium tuberculosis immunity. J Infect Dis 186, 1448–1457, doi: 10.1086/344359 (2002). 12404160

[b35] Fletcher H. A. . Inhibition of mycobacterial growth *in vitro* following primary but not secondary vaccination with Mycobacterium bovis BCG. Clin Vaccine Immunol 20, 1683–1689, doi: 10.1128/CVI.00427-13 (2013). 23986316 PMC3837779

[b36] Lancioni C. . CD8+ T cells provide an immunologic signature of tuberculosis in young children. Am J Respir Crit Care Med 185, 206–212, doi: 10.1164/rccm.201107-1355OC (2012). 22071329 PMC3297089

[b37] Lewinsohn D. M. . Human CD8 T Cell Antigens/Epitopes Identified by a Proteomic Peptide Library. PLoS One 8, e67016, doi: 10.1371/journal.pone.0067016 (2013). 23805289 PMC3689843

[b38] Lindenstrom T., Aagaard C., Christensen D., Agger E. M. & Andersen P. High-frequency vaccine-induced CD8(+) T cells specific for an epitope naturally processed during infection with Mycobacterium tuberculosis do not confer protection. Eur J Immunol 44, 1699–1709, doi: 10.1002/eji.201344358 (2014). 24677089 PMC4112357

[b39] Bennekov T. . Alteration of epitope recognition pattern in Ag85B and ESAT-6 has a profound influence on vaccine-induced protection against Mycobacterium tuberculosis. Eur J Immunol 36, 3346–3355, doi: 10.1002/eji.200636128 (2006). 17109467

[b40] Aagaard C. S., Hoang T. T., Vingsbo-Lundberg C., Dietrich J. & Andersen P. Quality and vaccine efficacy of CD4+ T cell responses directed to dominant and subdominant epitopes in ESAT-6 from Mycobacterium tuberculosis. J Immunol 183, 2659–2668, doi: 10.4049/jimmunol.0900947 (2009). 19620314

[b41] Olsen A. W., Hansen P. R., Holm A. & Andersen P. Efficient protection against Mycobacterium tuberculosis by vaccination with a single subdominant epitope from the ESAT-6 antigen. Eur J Immunol 30, 1724–1732, doi: 10.1002/1521-4141(200006)30:6<1724::AID-IMMU1724>3.0.CO;2-A(2000 ). 10898510

[b42] Woodworth J. S. . Protective CD4 T cells targeting cryptic epitopes of Mycobacterium tuberculosis resist infection-driven terminal differentiation. J Immunol 192, 3247–3258, doi: 10.4049/jimmunol.1300283 (2014). 24574499

[b43] Venturi V., Price D. A., Douek D. C. & Davenport M. P. The molecular basis for public T-cell responses? Nat Rev Immunol 8, 231–238, doi: 10.1038/nri2260 (2008). 18301425

[b44] Romani N. . Proliferating dendritic cell progenitors in human blood. J Exp Med 180, 83–93 (1994). 8006603 10.1084/jem.180.1.83 PMC2191538

