# Supplementary material for: Adenovirally-Induced Polyfunctional T Cells Do Not Necessarily Recognize the Infected Target: Lessons from a Phase I Trial of the AERAS-402 Vaccine
Source: Sci Rep. 2016 Nov 2;6:36355. doi: 10.1038/srep36355 (PMC5141283; doi:10.1038/srep36355)
Supplement: Supplementary Information [file srep36355-s1.pdf]

Supplementary Figures / Table:

Adenovirally-Induced Polyfunctional T Cells Do Not Necessarily Recognize the Infected Target: Lessons from a Phase I Trial of the AERAS-402 Vaccine

Authors: Melissa Nyendak [1*], Gwendolyn M. Swarbrick [2,3], Amanda Duncan [2], Meghan Cansler[2] Ervina Winata Huff [3], David Hokey [4], Tom Evans [4], Lewellys Barker [4], Gretta Blatner [4], Jerald Sadoff [5], Macaya Douoguih [5], Maria Grazia Pau [5], Deborah A. Lewinsohn [2], David M. Lewinsohn [1,2,3*]

Affiliations:

[1] Department of Medicine, Oregon Health and Science University, Portland, Oregon, USA.

[2] Department of Pediatrics, Oregon Health and Science University, Portland, Oregon, USA.

[3]Department of Medicine, Portland VA Medical Center, Portland, Oregon, USA.

[4]Aeras, Rockville, Maryland, USA.

[5]Janssen Infectious Diseases and Vaccines (formerly Crucell), Leiden, The Netherlands.

*Corresponding authors. Email: nyendakm@ohsu.edu, lewinsod@ohsu.edu

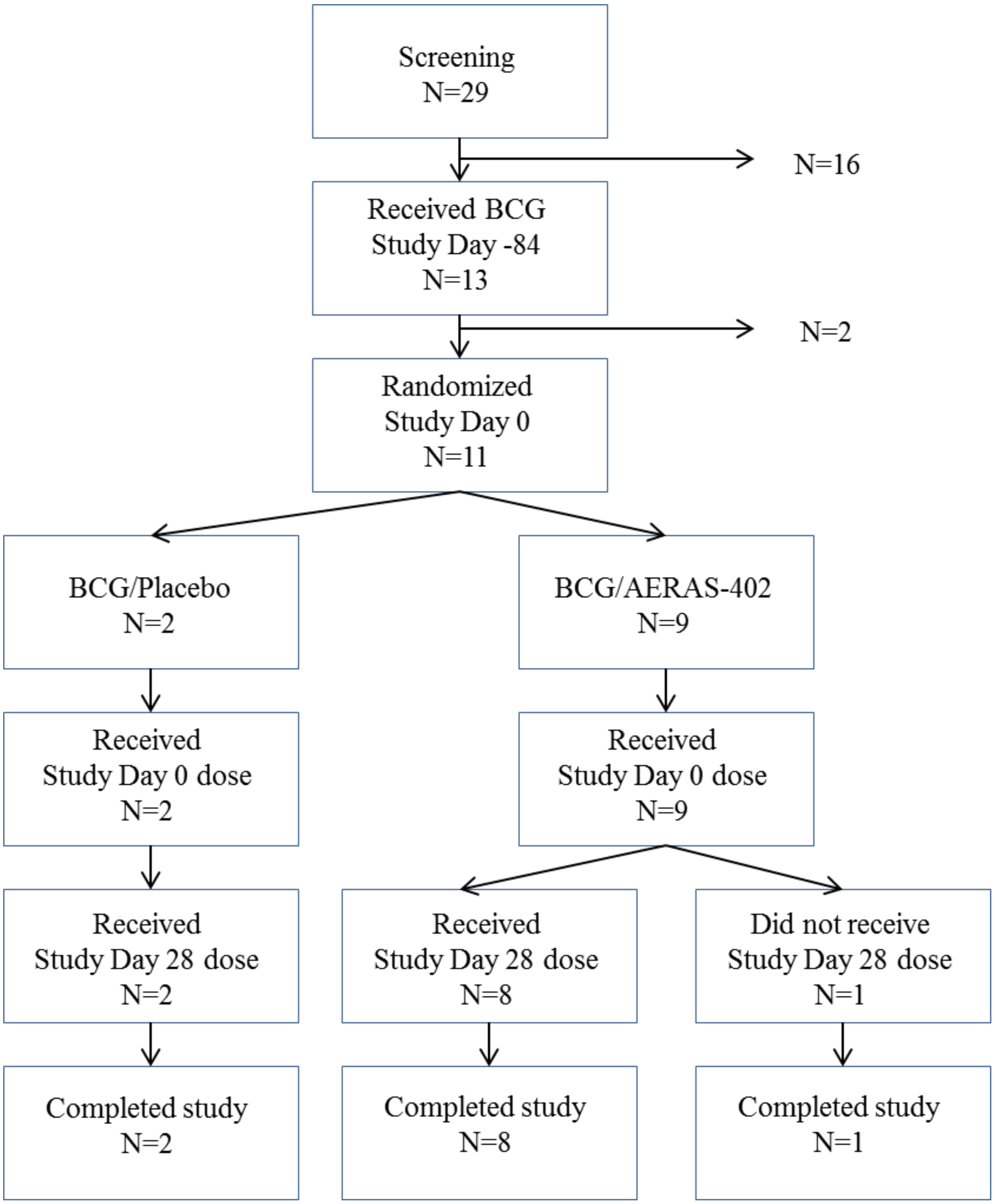

Supplementary Figure S1. Consort Diagram. All participants received BCG on day -84 and 9 participants received AERAS-402 on day 0 and 8 participants received AERAS-402 on day 28. Two participants received placebo on day 0 and day 28.

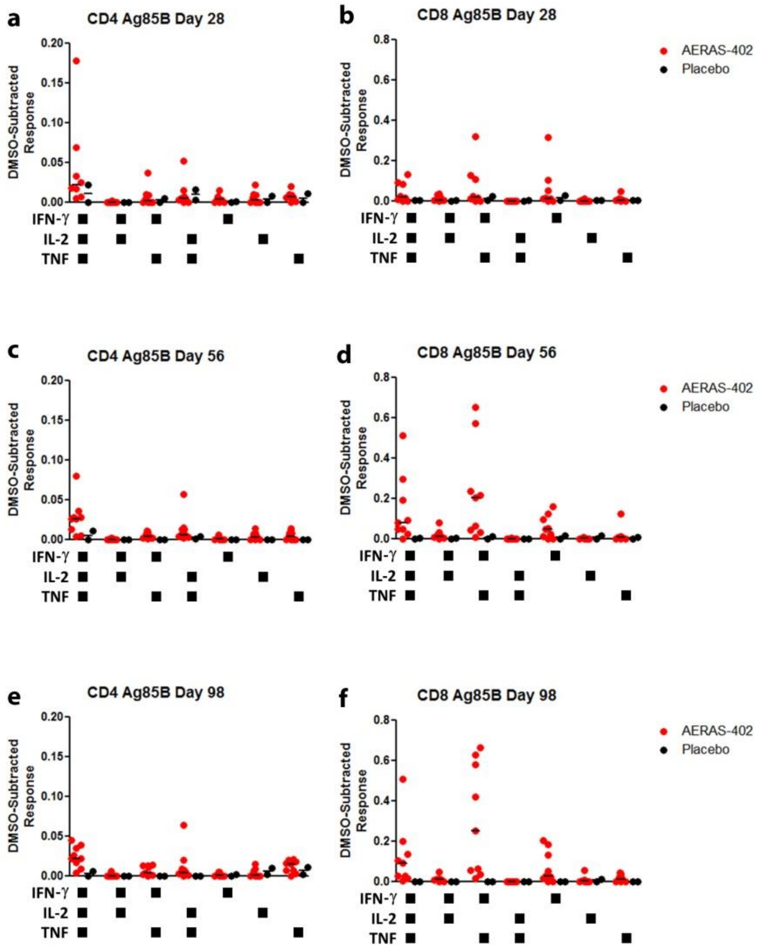

Supplementary Figure S2. Polyfunctional Analysis on Study Days 28, 56 and 98. PBMC and leukapheresis specimens from study days 28 (a, b), 56 (c, d) and 98 (e, f) were thawed, rested overnight, and stimulated for 5-7 hours with DMSO (negative control), SEB (positive control), or the Ag85B peptide pool. Specimens were then stained for viability, phenotypic markers, and intracellular cytokine expression and evaluated by flow cytometry. Data were analyzed using FlowJo software to generate cytokine Boolean gates. Each gate was subjected to DMSO subtraction to remove background and plotted as percent response of CD4 (a, c, e) or CD8 (b, d, f) T cell populations. Each circle represents the response from a single participant. Bars represent the median response for each group. Data is shown for participants immunized with BCG on Study Day -84 and placebo (black circles) and participants vaccinated with BCG on Study Day -84 followed by vaccination with AERAS-402 on Study Days 0 and 28 ($3x10^{10}$ vp; red circles). The gating strategy is shown in Figure 2, panel a.

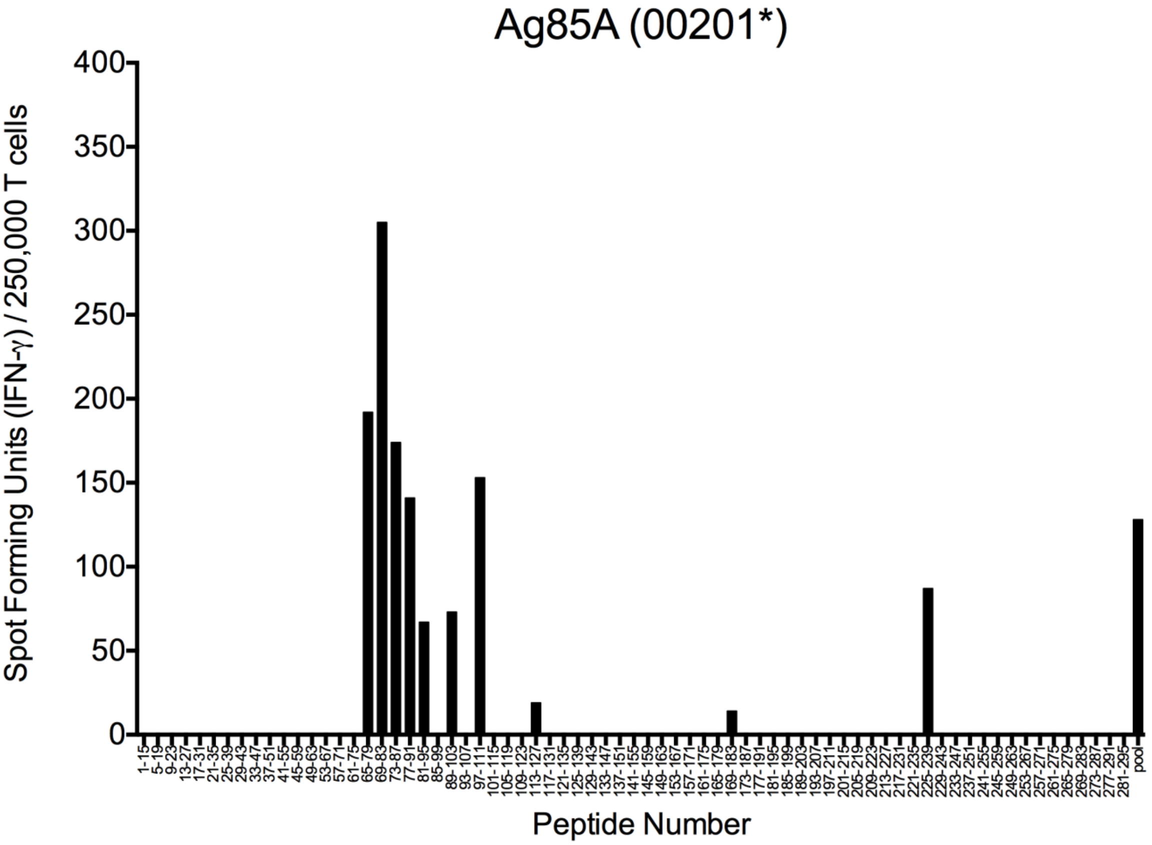
Ag85A (00201*)
Spot Forming Units (IFN-γ) / 250,000 T cells
400
350
300
250
200
150
100
50
0
Peptide Number

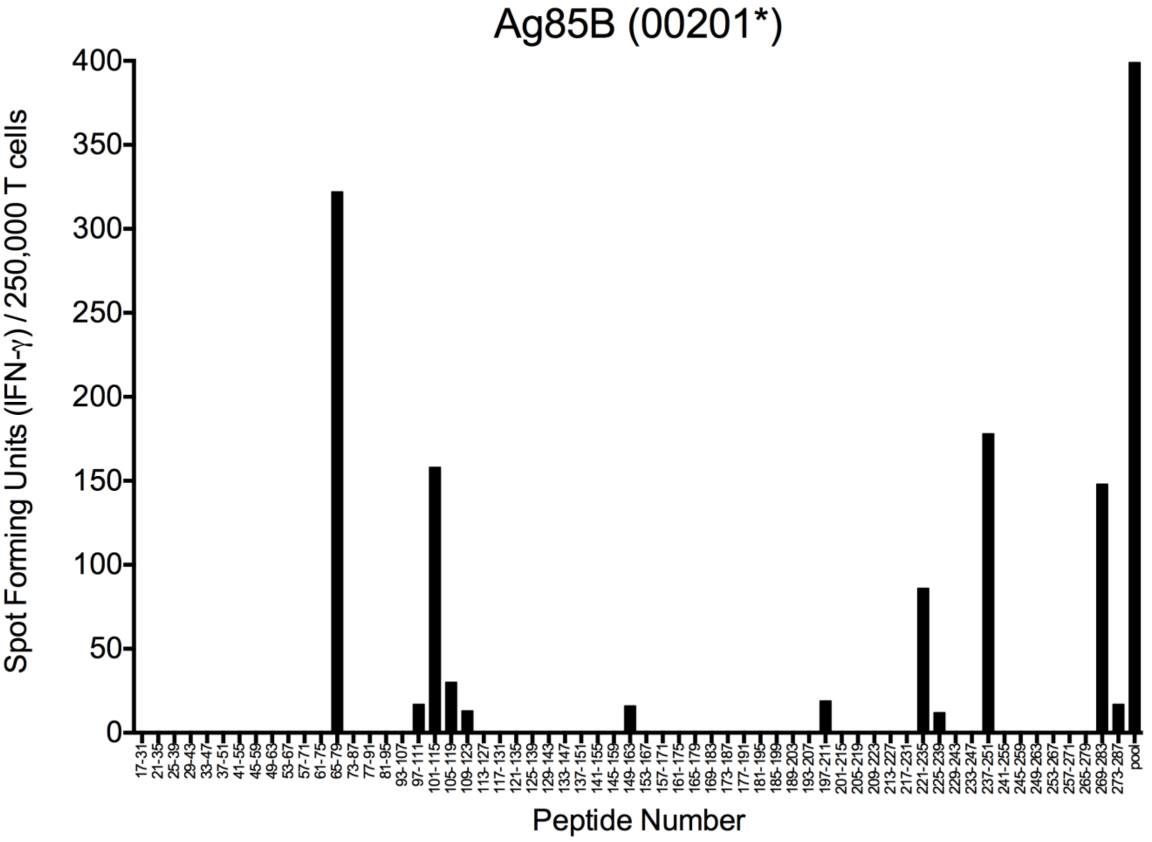
Ag85B (00201*)
Spot Forming Units (IFN-γ) / 250,000 T cells
400
350
300
250
200
150
100
50
0
Peptide Number

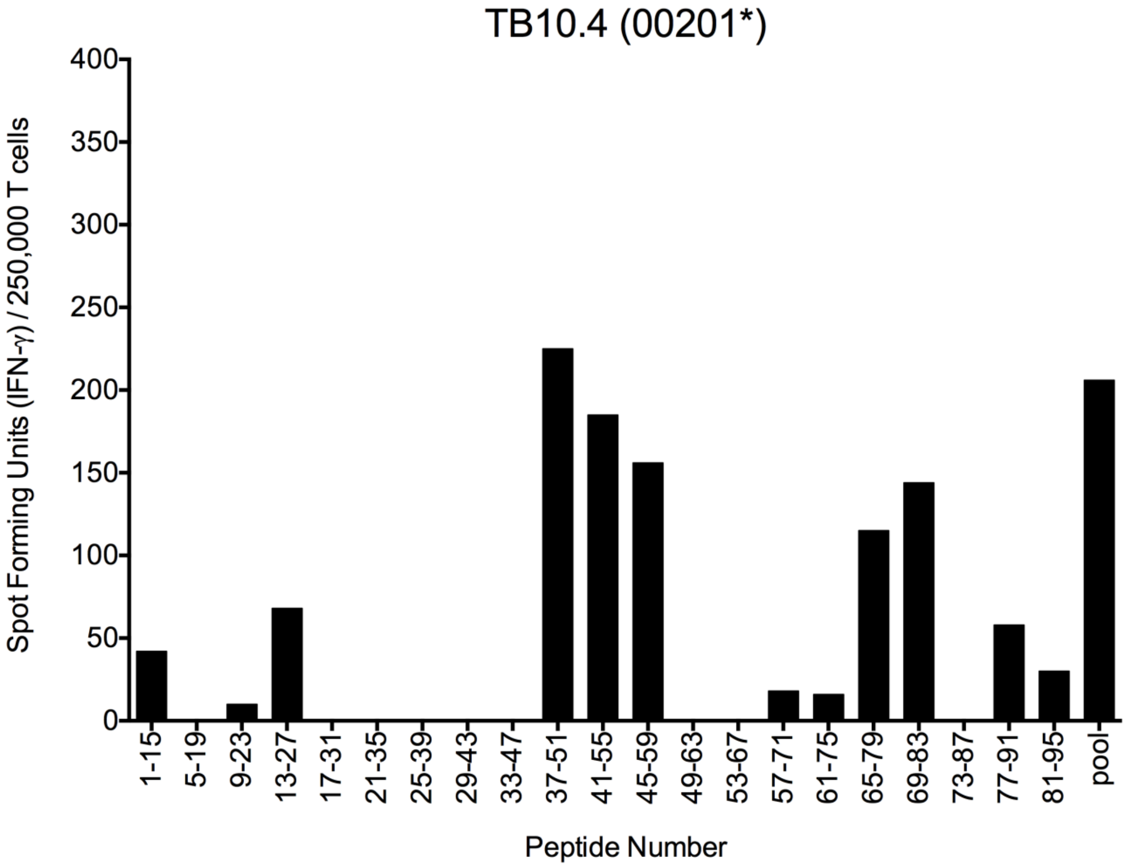
TB10.4 (00201*)
Spot Forming Units (IFN-γ) / 250,000 T cells
400
350
300
250
200
150
100
50
0
1-15
5-19
9-23
13-27
17-31
21-35
25-39
29-43
33-47
37-51
41-55
45-59
49-63
53-67
57-71
61-75
65-79
69-83
73-87
77-91
81-95
pool
Peptide Number

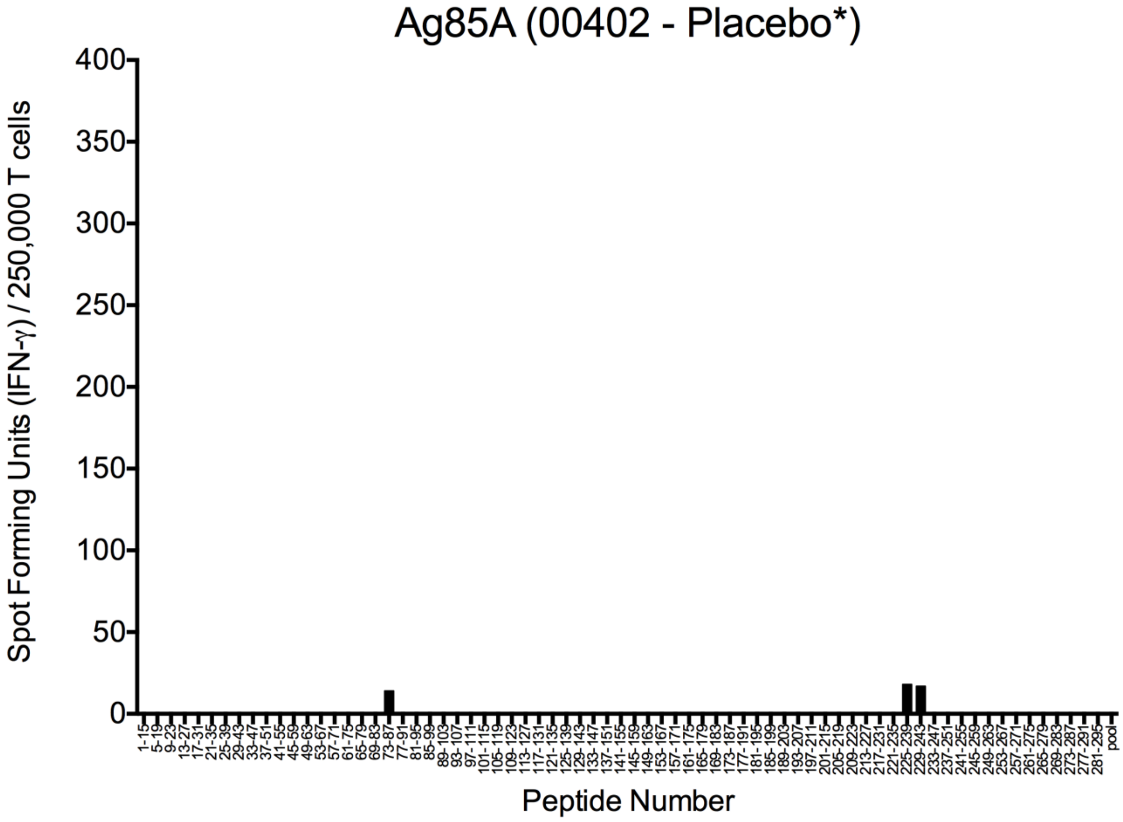

Ag85A (00402 - Placebo*)
Spot Forming Units (IFN-γ) / 250,000 T cells
Peptide Number

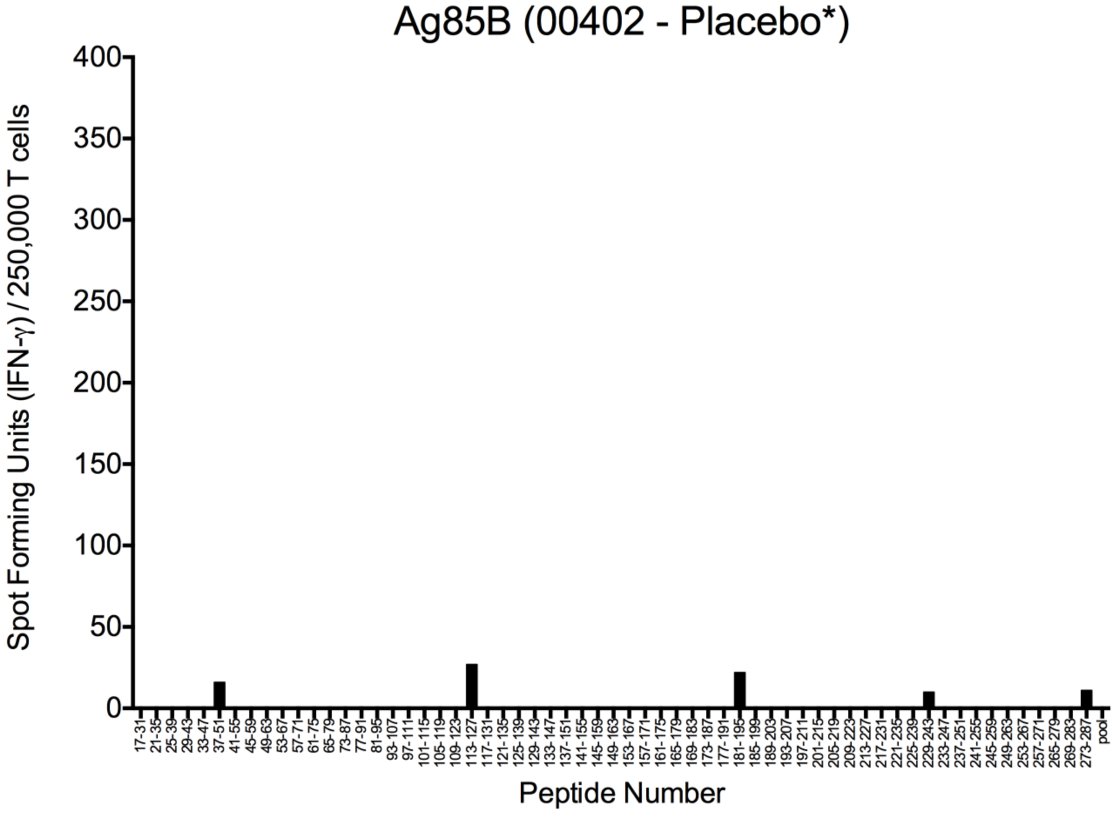

Ag85B (00402 - Placebo*)
Spot Forming Units (IFN-γ) / 250,000 T cells
Peptide Number

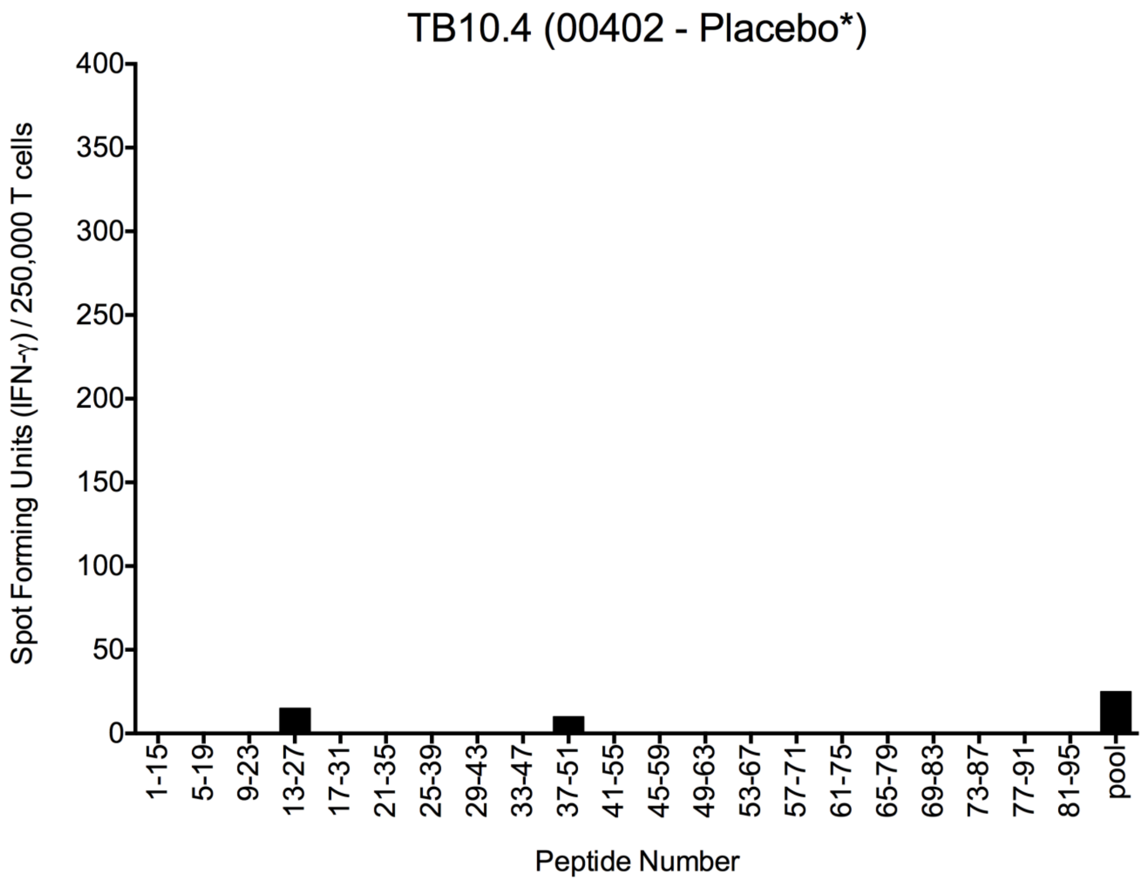
TB10.4 (00402 - Placebo*)
Spot Forming Units (IFN-γ) / 250,000 T cells
400
350
300
250
200
150
100
50
0
1-15
5-19
9-23
13-27
17-31
21-35
25-39
29-43
33-47
37-51
41-55
45-59
49-63
53-67
57-71
61-75
65-79
69-83
73-87
77-91
81-95
pool
Peptide Number

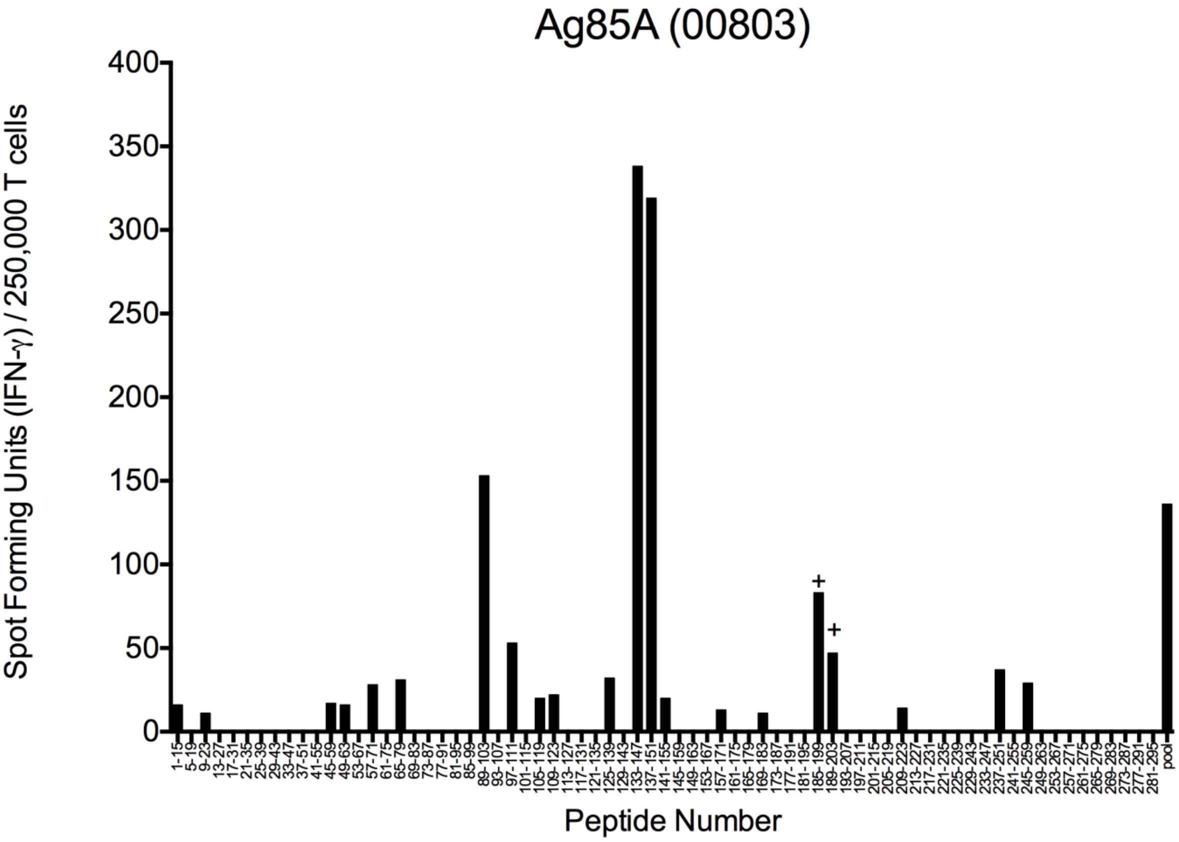
Ag85A (00803)
Spot Forming Units (IFN-γ) / 250,000 T cells
400
350
300
250
200
150
100
50
0
+
+
Peptide Number

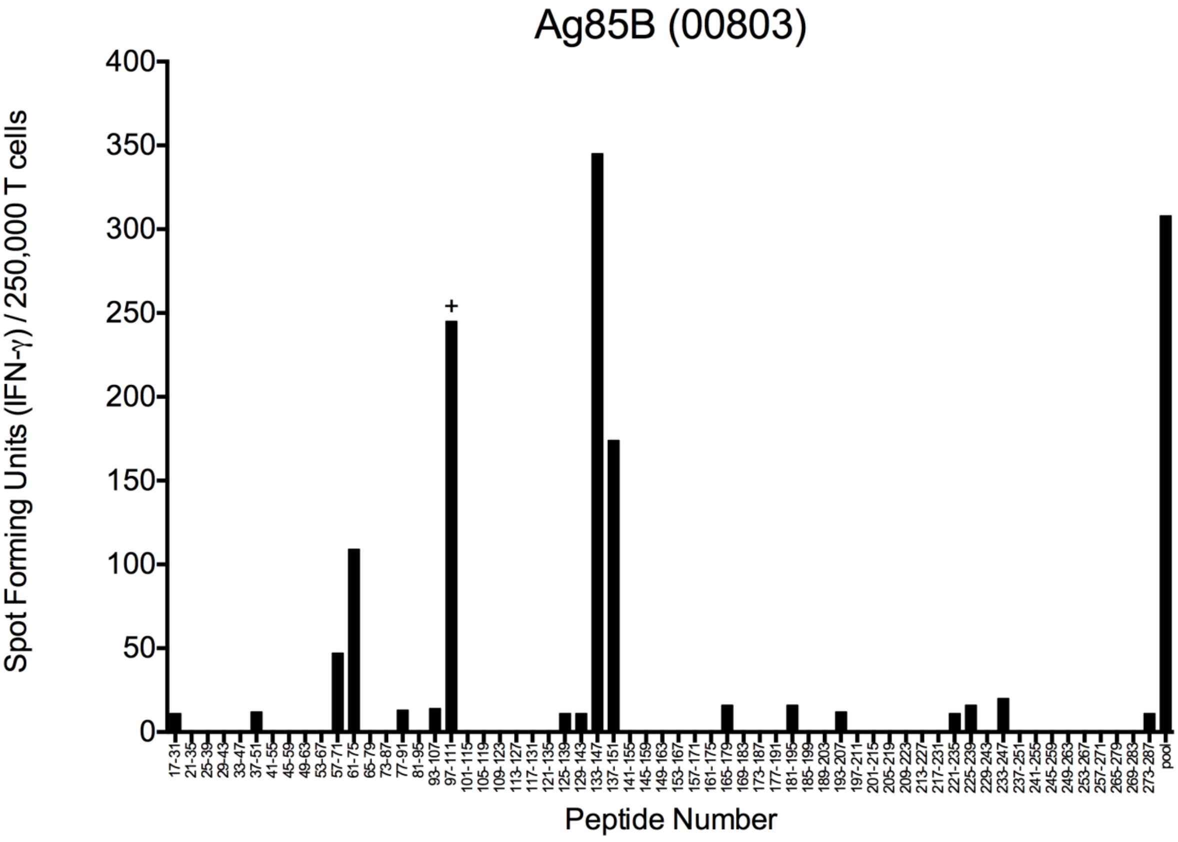
Ag85B (00803)
Spot Forming Units (IFN-γ) / 250,000 T cells
400
350
300
250
200
150
100
50
0
+
Peptide Number

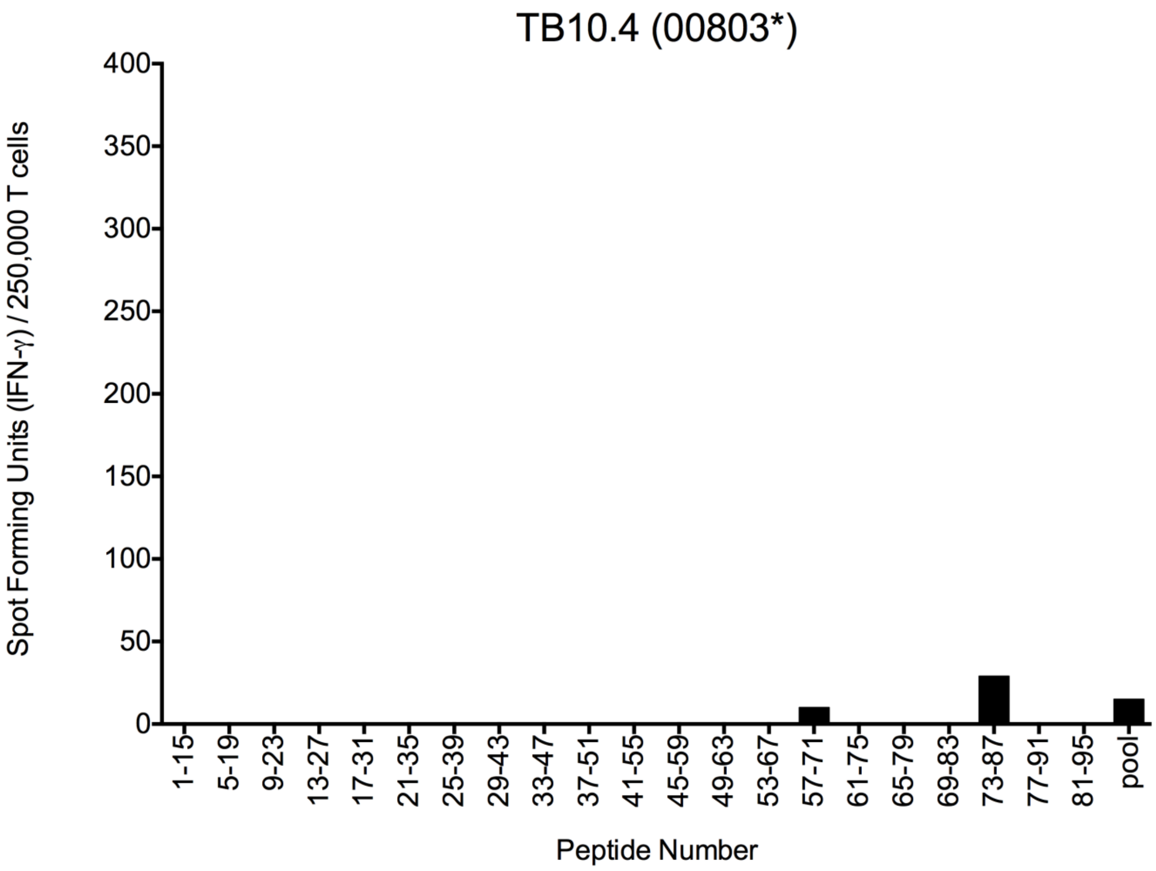

TB10.4 (00803*)
Spot Forming Units (IFN-γ) / 250,000 T cells
400
350
300
250
200
150
100
50
0
1-15
5-19
9-23
13-27
17-31
21-35
25-39
29-43
33-47
37-51
41-55
45-59
49-63
53-67
57-71
61-75
65-79
69-83
73-87
77-91
81-95
pool
Peptide Number

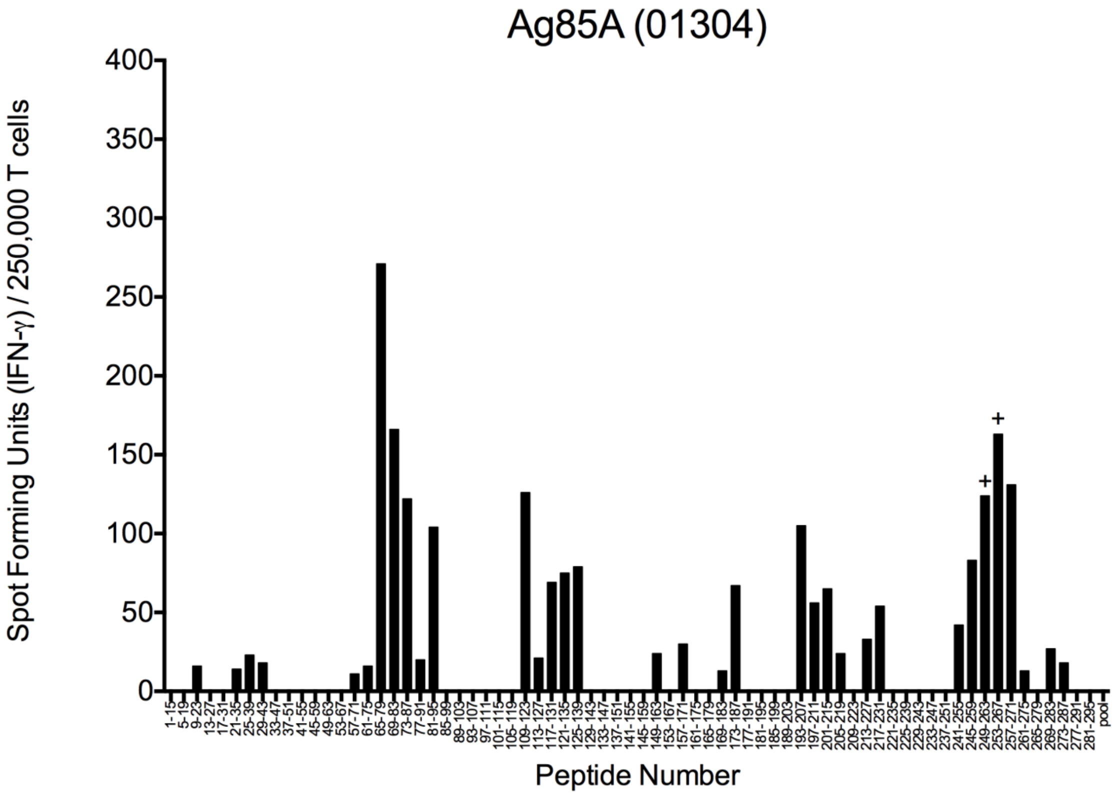
Ag85A (01304)
Spot Forming Units (IFN-γ) / 250,000 T cells
400
350
300
250
200
150
100
50
0
+
+
1-15 5-19 9-23 13-27 17-31 21-35 25-39 29-43 33-47 37-51 41-55 45-59 49-63 53-67 57-71 61-75 65-79 69-83 73-87 77-91 81-95 85-99 89-103 93-107 97-111 101-115 105-119 109-123 113-127 117-131 121-135 125-139 129-143 133-147 137-151 141-155 145-159 149-163 153-167 157-171 161-175 165-179 169-183 173-187 177-191 181-195 185-199 189-203 193-207 197-211 201-215 205-219 209-223 213-227 217-231 221-235 225-239 229-243 233-247 237-251 241-255 245-259 249-263 253-267 257-271 261-275 265-279 269-283 273-287 277-291 281-295 pool
Peptide Number

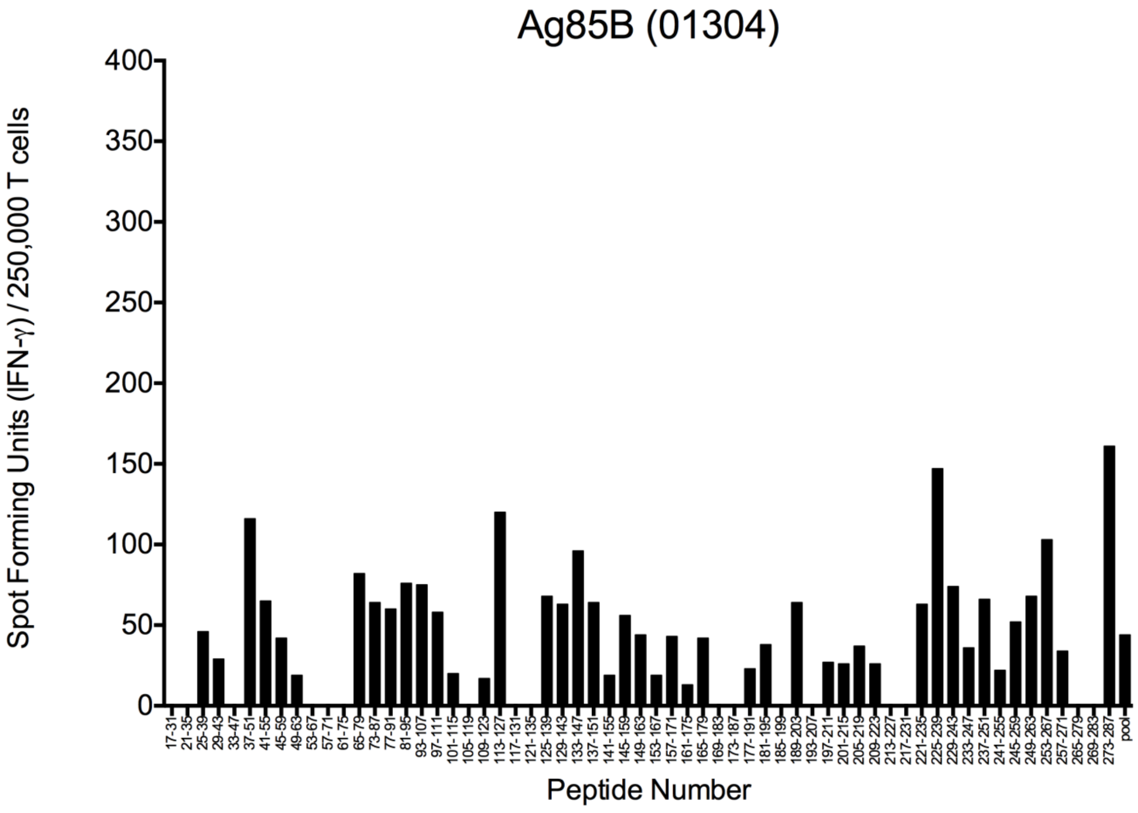
Ag85B (01304)
Spot Forming Units (IFN-γ) / 250,000 T cells
400
350
300
250
200
150
100
50
0
17-31 21-35 25-39 29-43 33-47 37-51 41-55 45-59 49-63 53-67 57-71 61-75 65-79 73-87 77-91 81-95 93-107 97-111 101-115 105-119 109-123 113-127 117-131 121-135 125-139 129-143 133-147 137-151 141-155 145-159 149-163 153-167 157-171 161-175 165-179 169-183 173-187 177-191 181-195 185-199 189-203 193-207 197-211 201-215 205-219 209-223 213-227 217-231 221-235 225-239 229-243 233-247 237-251 241-255 245-259 249-263 253-267 257-271 265-279 269-283 273-287 pool
Peptide Number

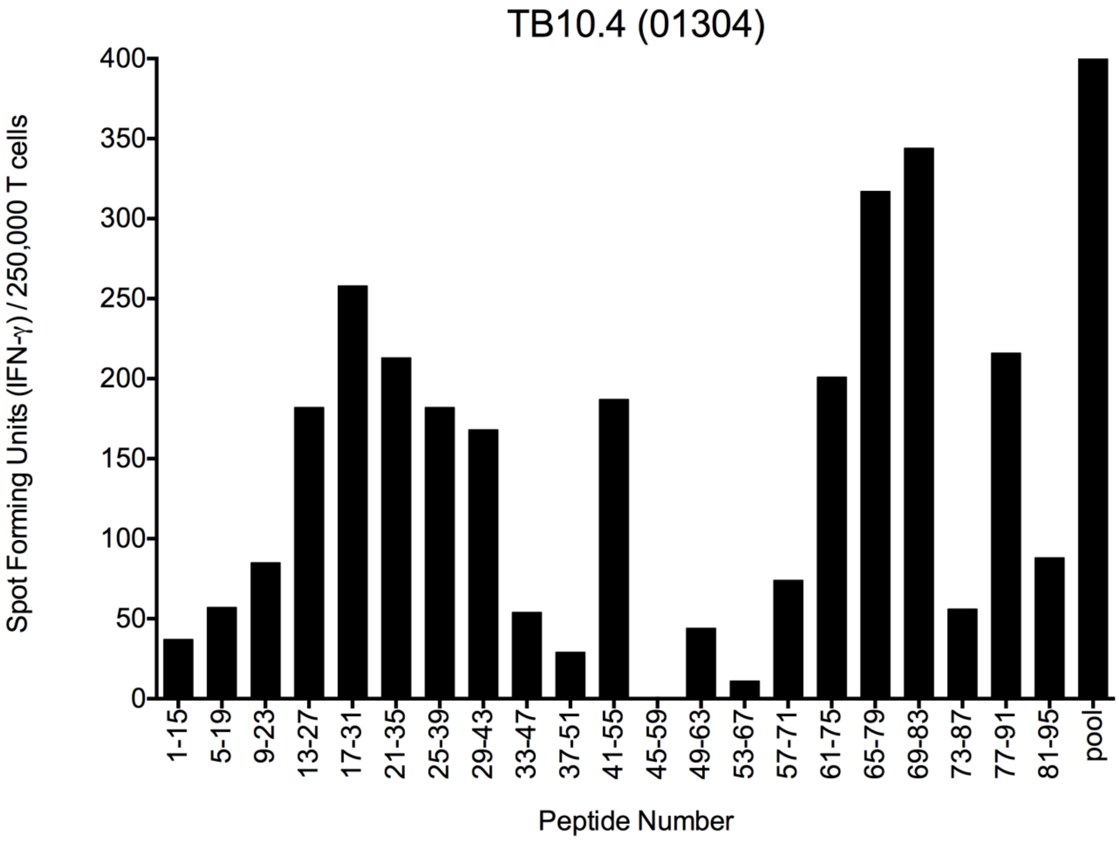
TB10.4 (01304)
Spot Forming Units (IFN-γ) / 250,000 T cells
400
350
300
250
200
150
100
50
0
1-15
5-19
9-23
13-27
17-31
21-35
25-39
29-43
33-47
37-51
41-55
45-59
49-63
53-67
57-71
61-75
65-79
69-83
73-87
77-91
81-95
pool
Peptide Number

Ag85A (01706*)

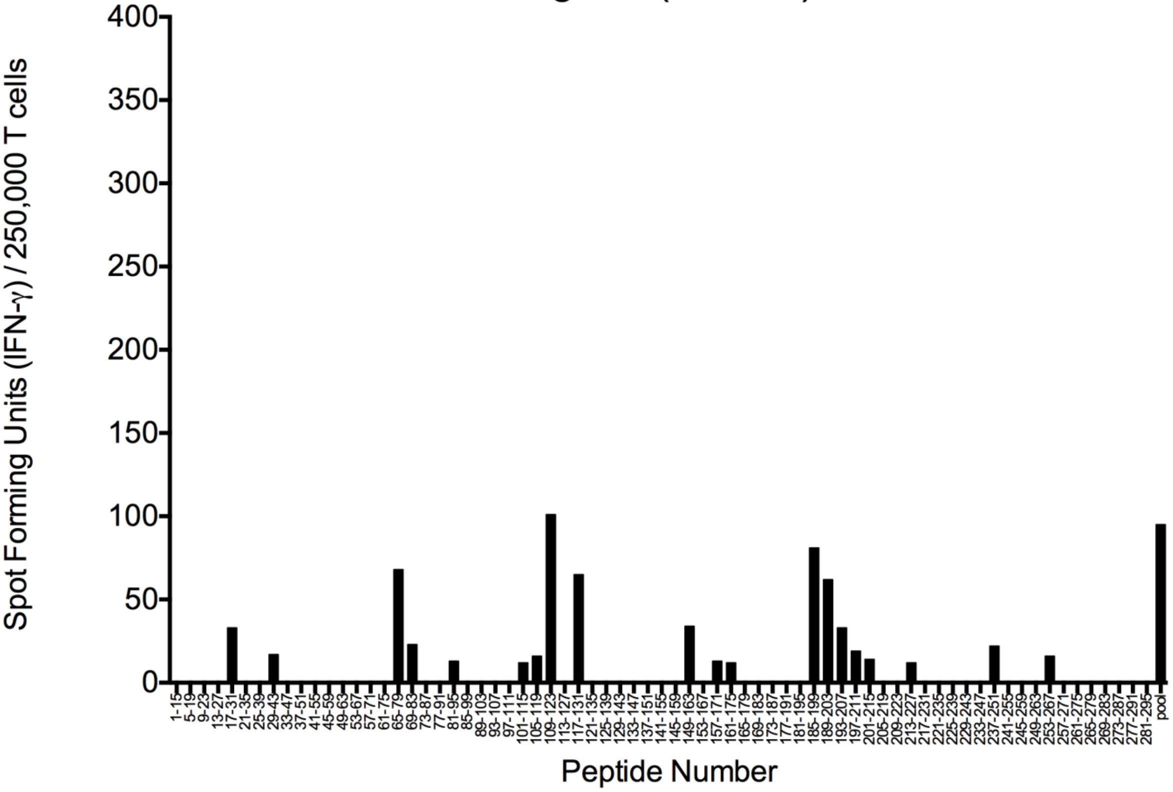

Ag85B (01706*)

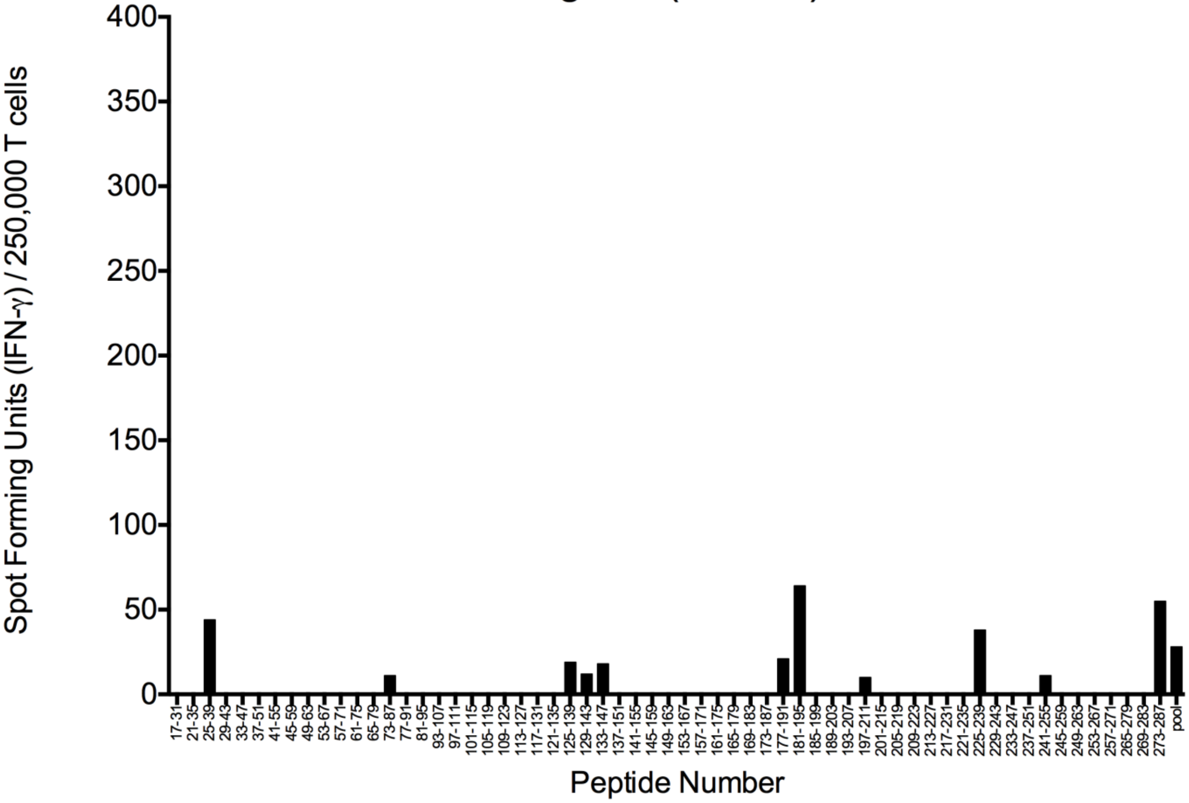

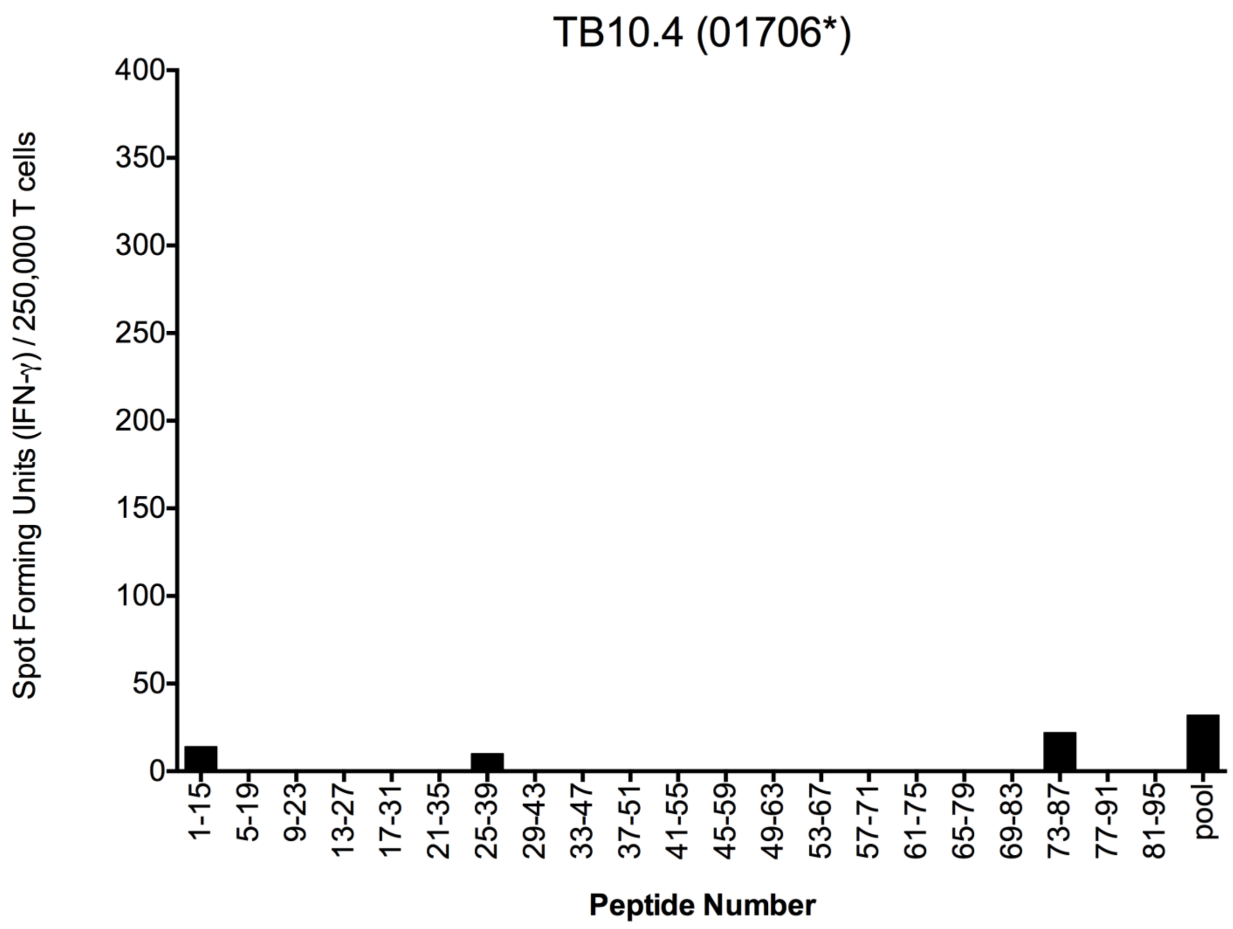
TB10.4 (01706*)
Spot Forming Units (IFN-γ) / 250,000 T cells
400
350
300
250
200
150
100
50
0
1-15
5-19
9-23
13-27
17-31
21-35
25-39
29-43
33-47
37-51
41-55
45-59
49-63
53-67
57-71
61-75
65-79
69-83
73-87
77-91
81-95
pool
Peptide Number

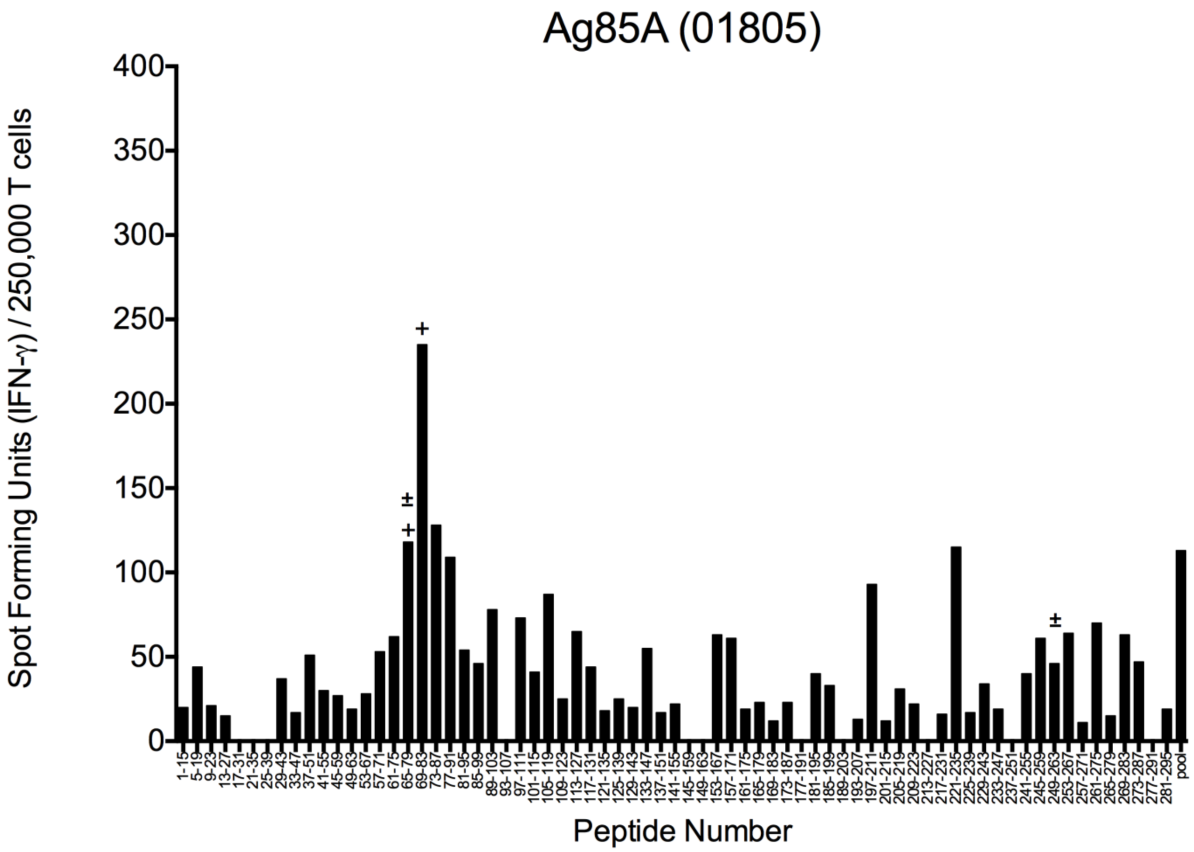

Ag85B (01805): insufficient cells

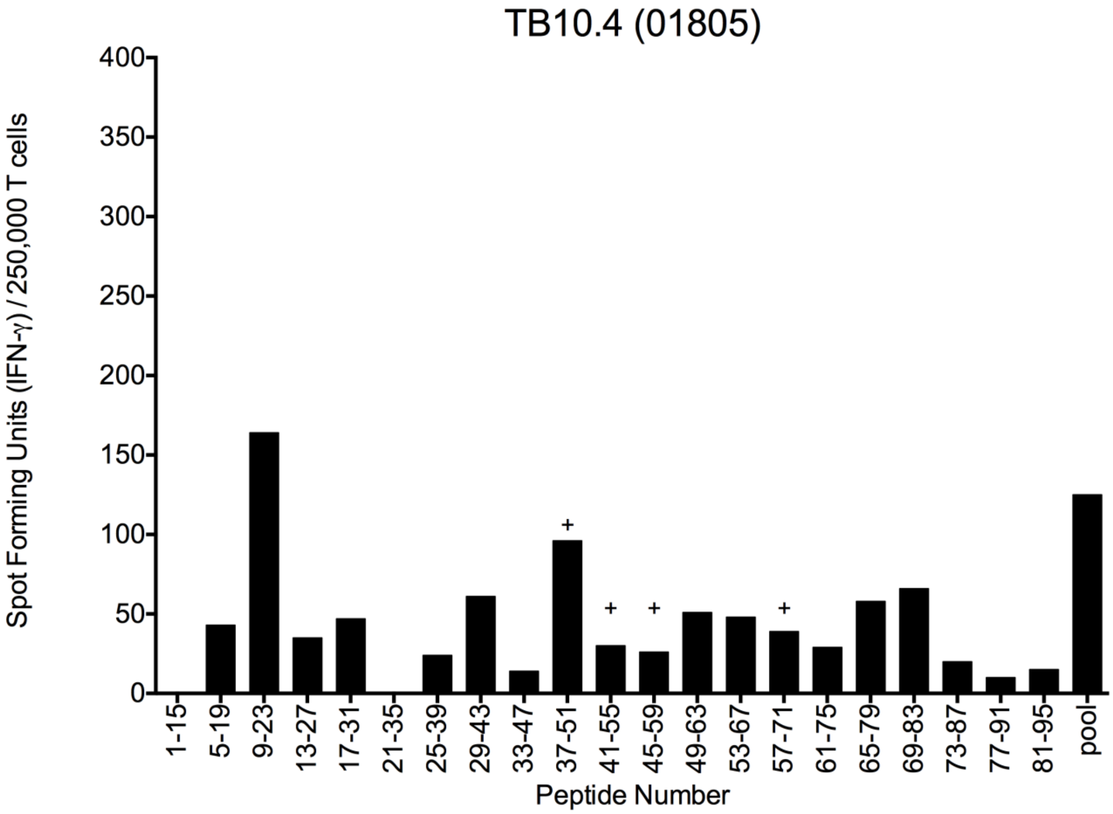

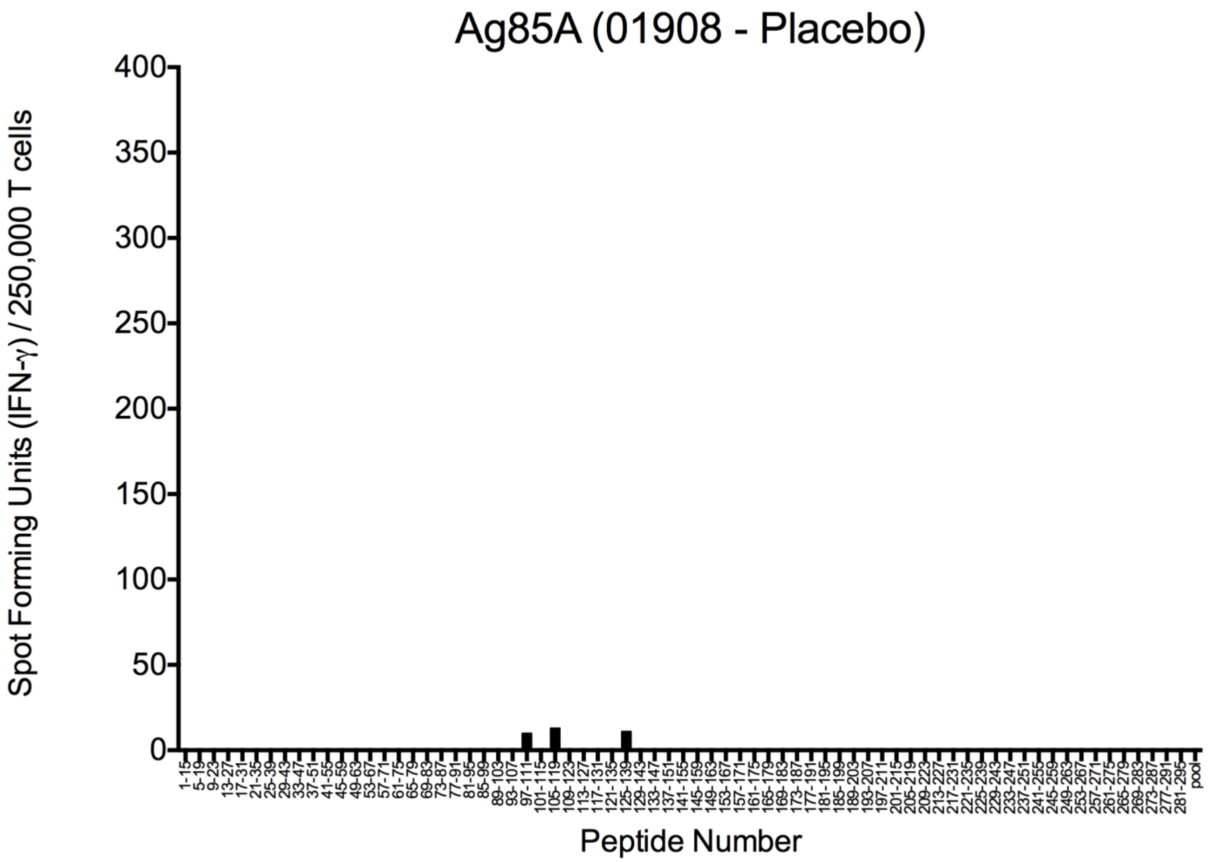
Ag85A (01908 - Placebo)
Spot Forming Units (IFN-γ) / 250,000 T cells
Peptide Number

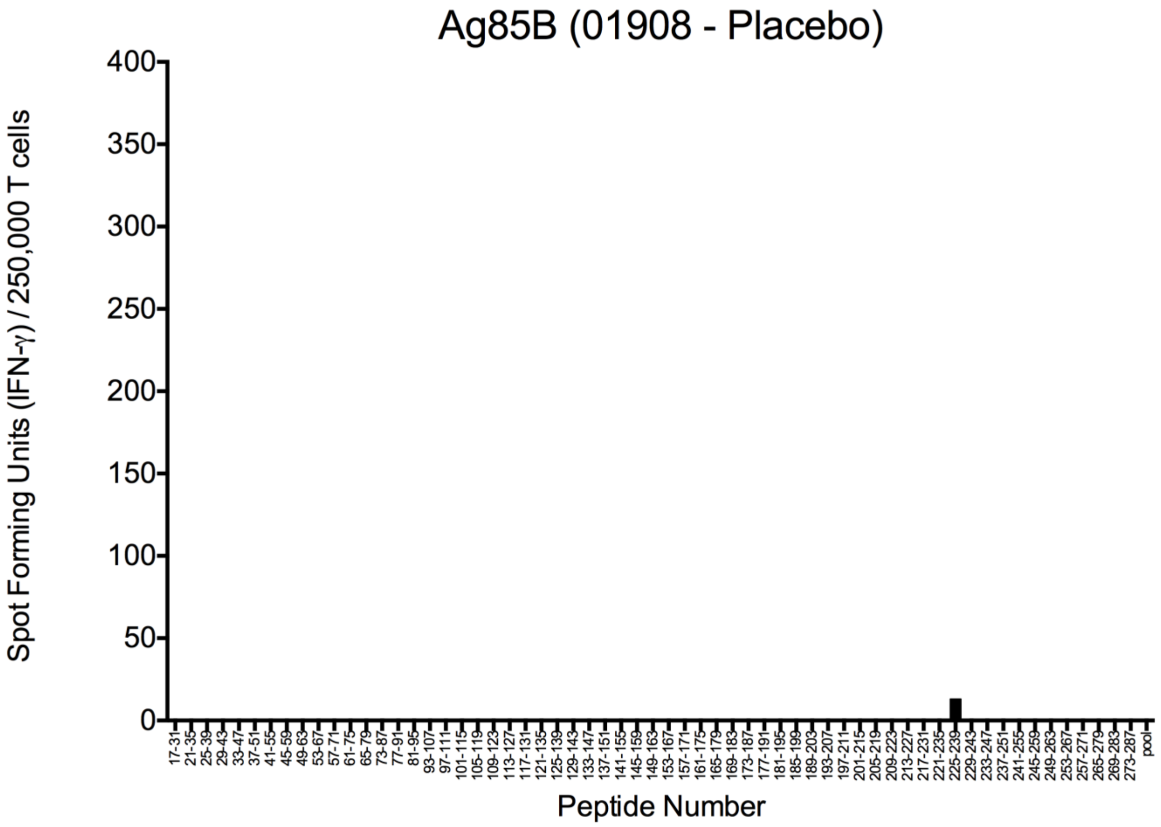
Ag85B (01908 - Placebo)
Spot Forming Units (IFN-γ) / 250,000 T cells
Peptide Number

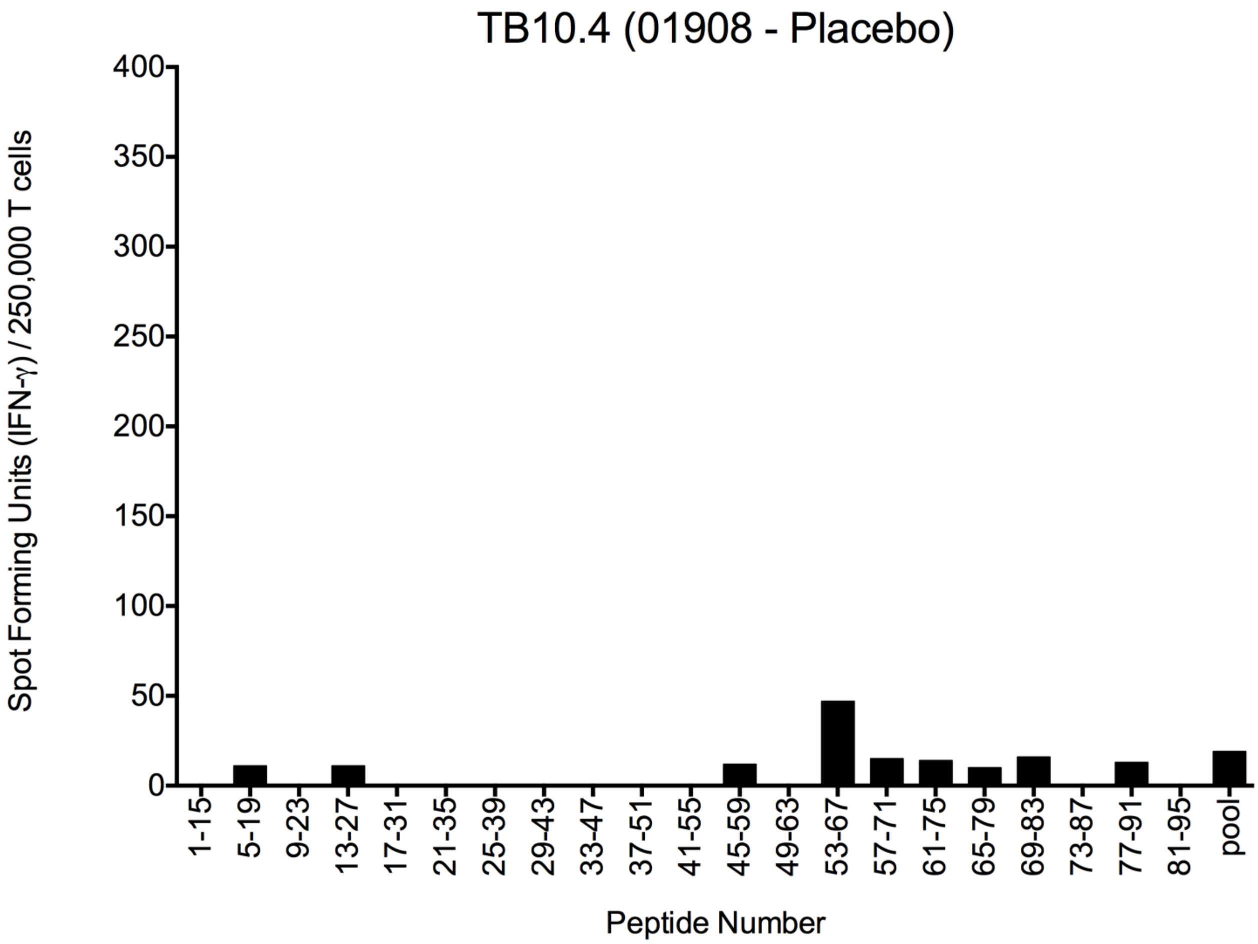
TB10.4 (01908 - Placebo)
Spot Forming Units (IFN-γ) / 250,000 T cells
400
350
300
250
200
150
100
50
0
1-15
5-19
9-23
13-27
17-31
21-35
25-39
29-43
33-47
37-51
41-55
45-59
49-63
53-67
57-71
61-75
65-79
69-83
73-87
77-91
81-95
pool
Peptide Number

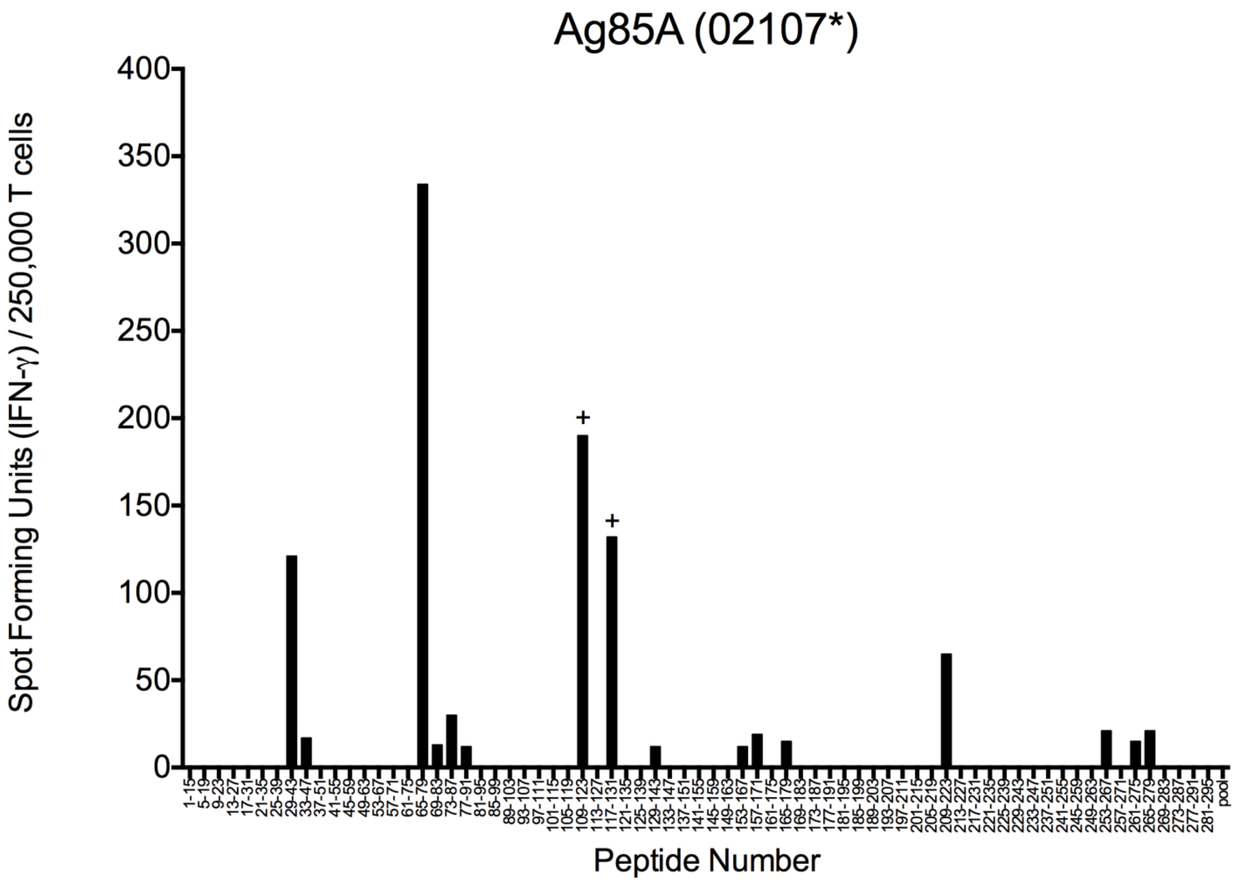
Ag85A (02107*)
Spot Forming Units (IFN-γ) / 250,000 T cells
Peptide Number

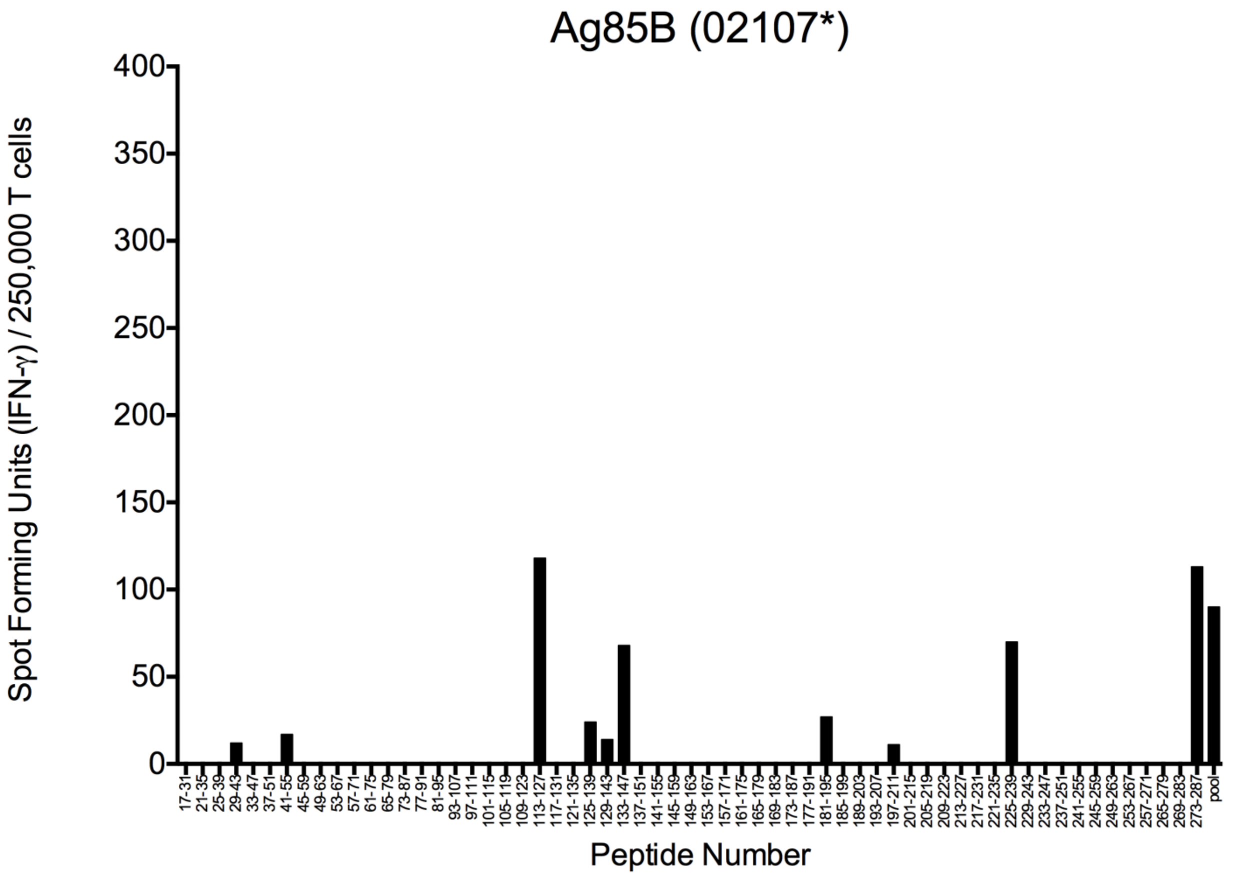
Ag85B (02107*)
Spot Forming Units (IFN-γ) / 250,000 T cells
Peptide Number

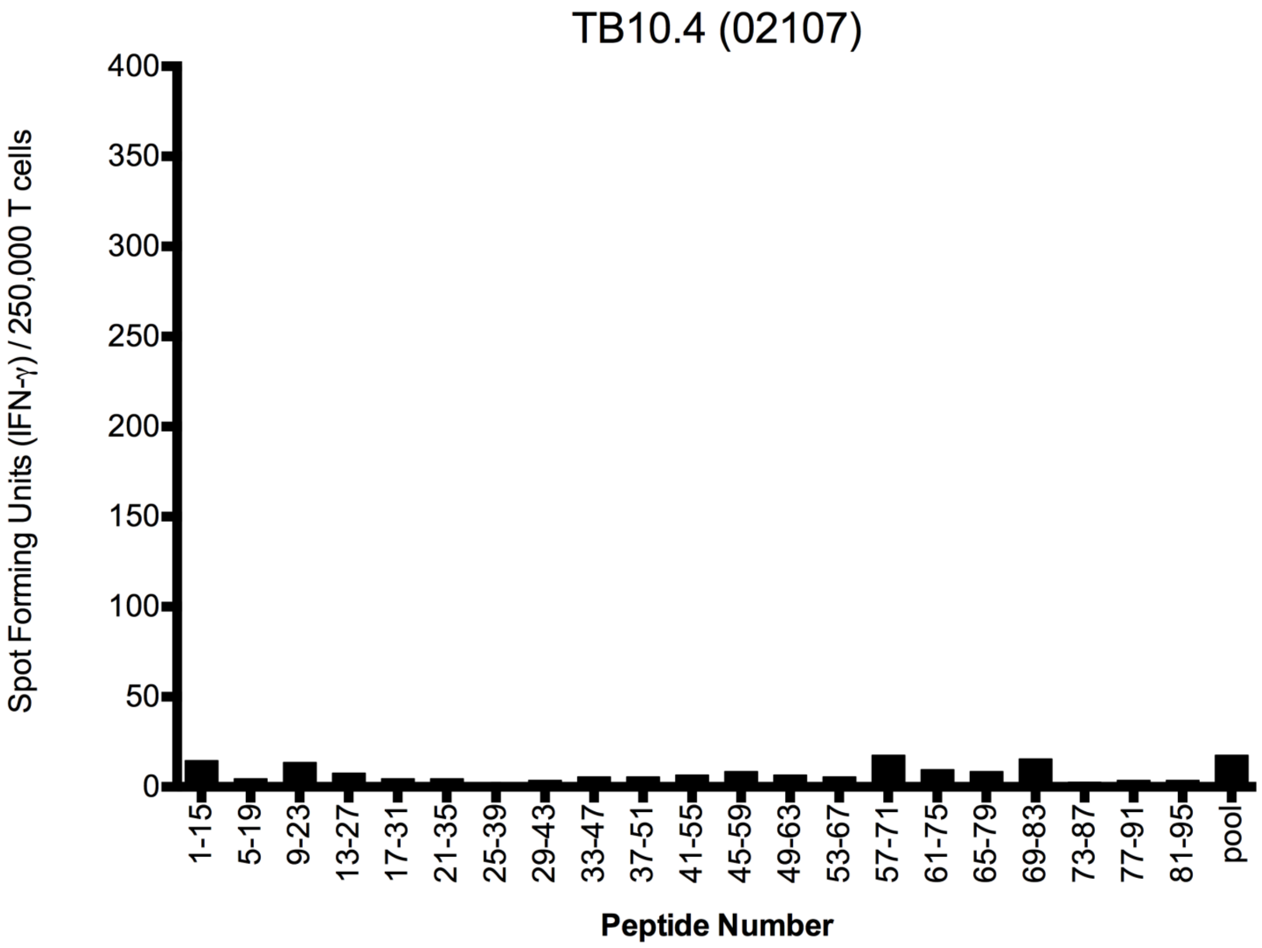
TB10.4 (02107)
Spot Forming Units (IFN-γ) / 250,000 T cells
400
350
300
250
200
150
100
50
0
1-15
5-19
9-23
13-27
17-31
21-35
25-39
29-43
33-47
37-51
41-55
45-59
49-63
53-67
57-71
61-75
65-79
69-83
73-87
77-91
81-95
pool
Peptide Number

Ag85A (02309)

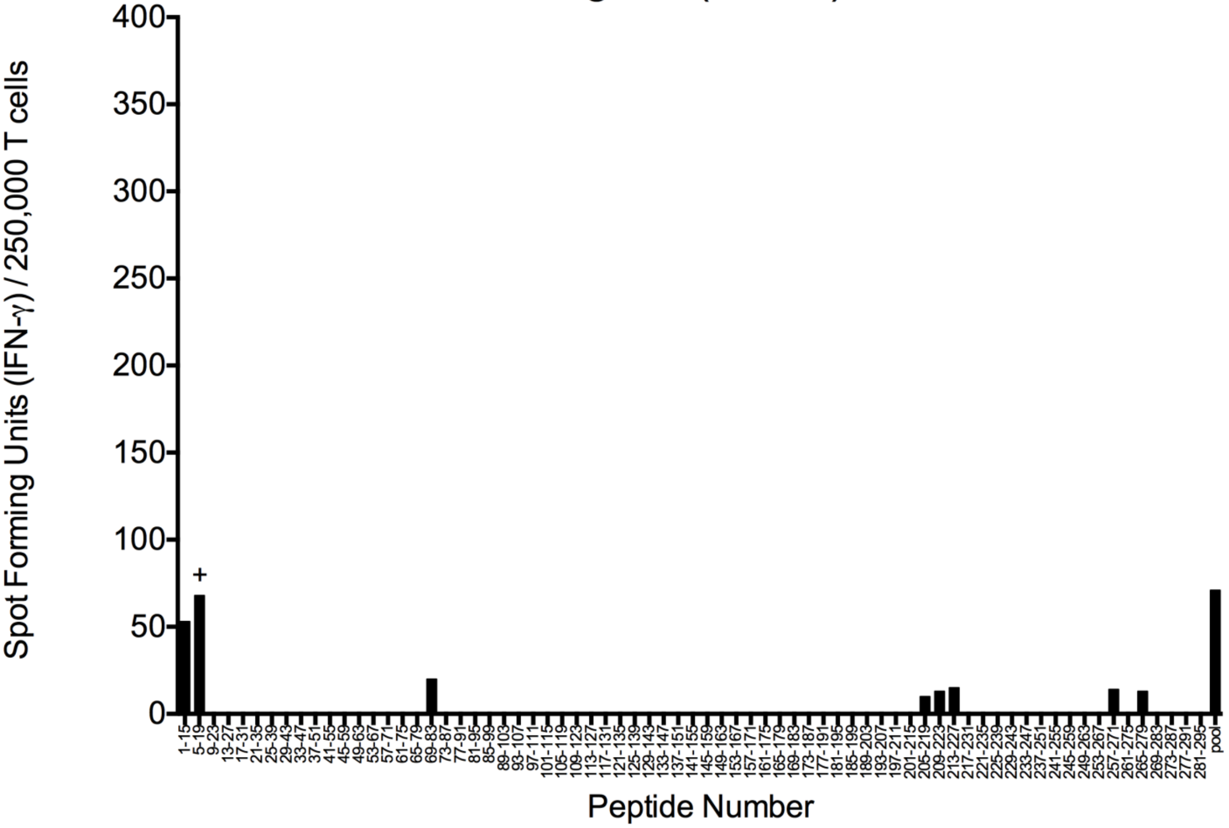

Ag85B (02309)

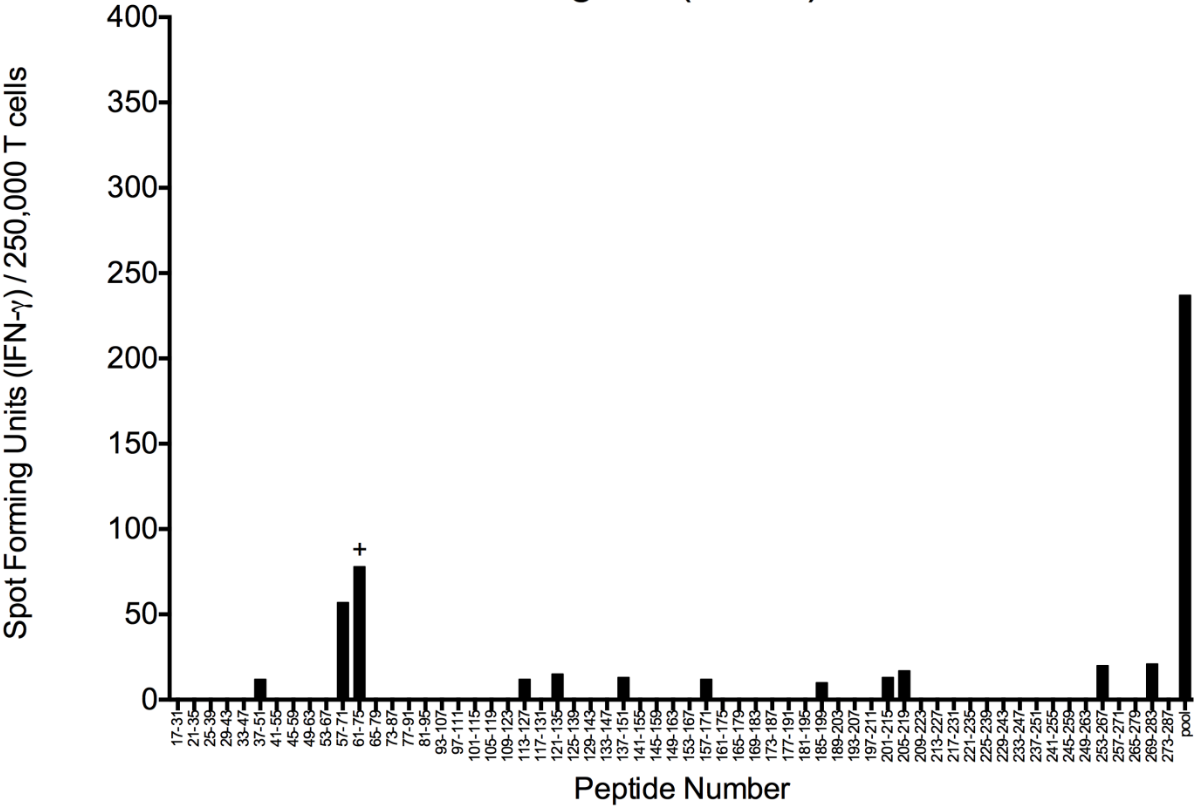

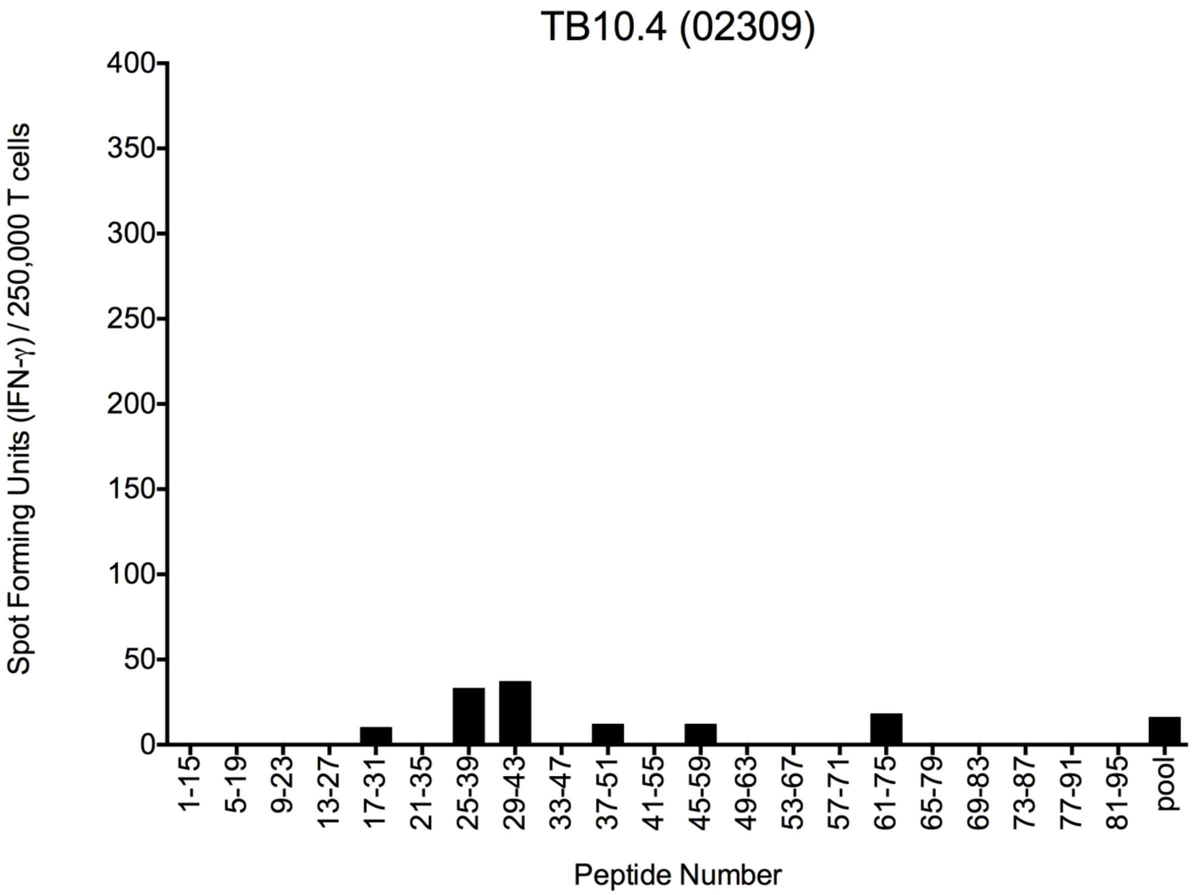
TB10.4 (02309)
Spot Forming Units (IFN-γ) / 250,000 T cells
400
350
300
250
200
150
100
50
0
1-15
5-19
9-23
13-27
17-31
21-35
25-39
29-43
33-47
37-51
41-55
45-59
49-63
53-67
57-71
61-75
65-79
69-83
73-87
77-91
81-95
pool
Peptide Number

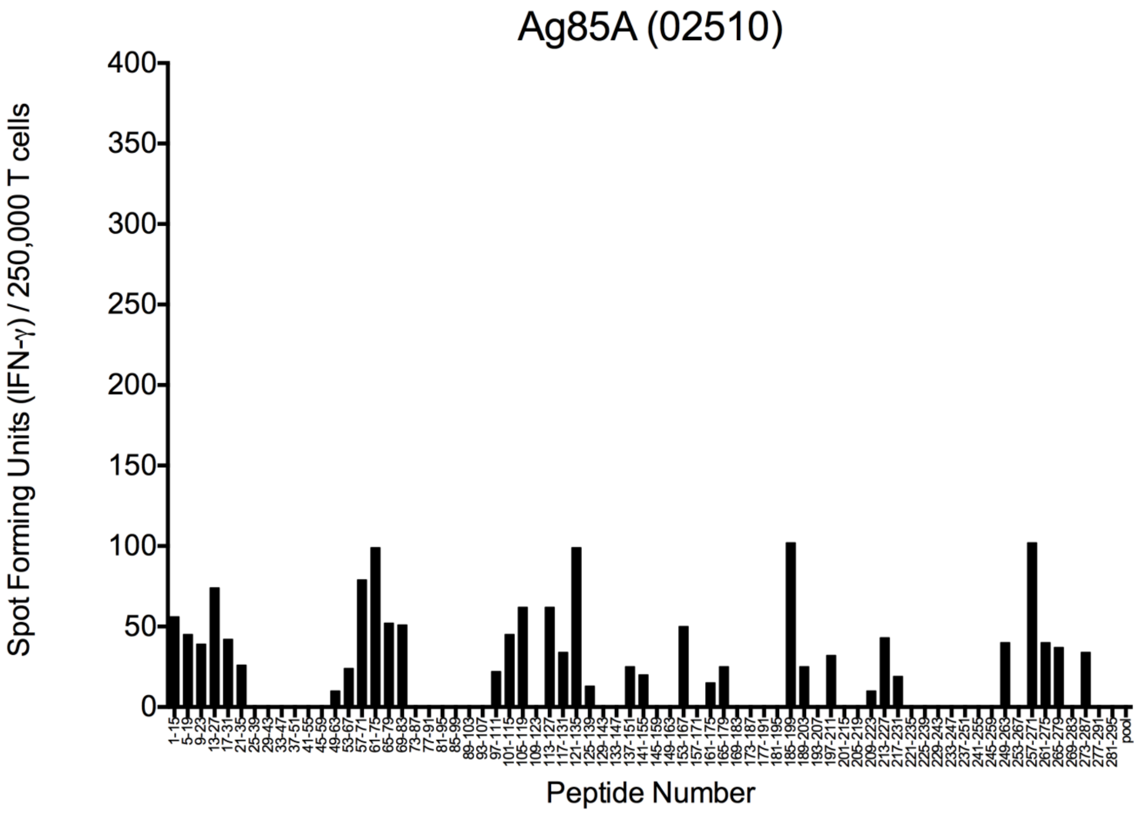
Ag85A (02510)
Spot Forming Units (IFN-γ) / 250,000 T cells
Peptide Number
0
50
100
150
200
250
300
350
400
pool

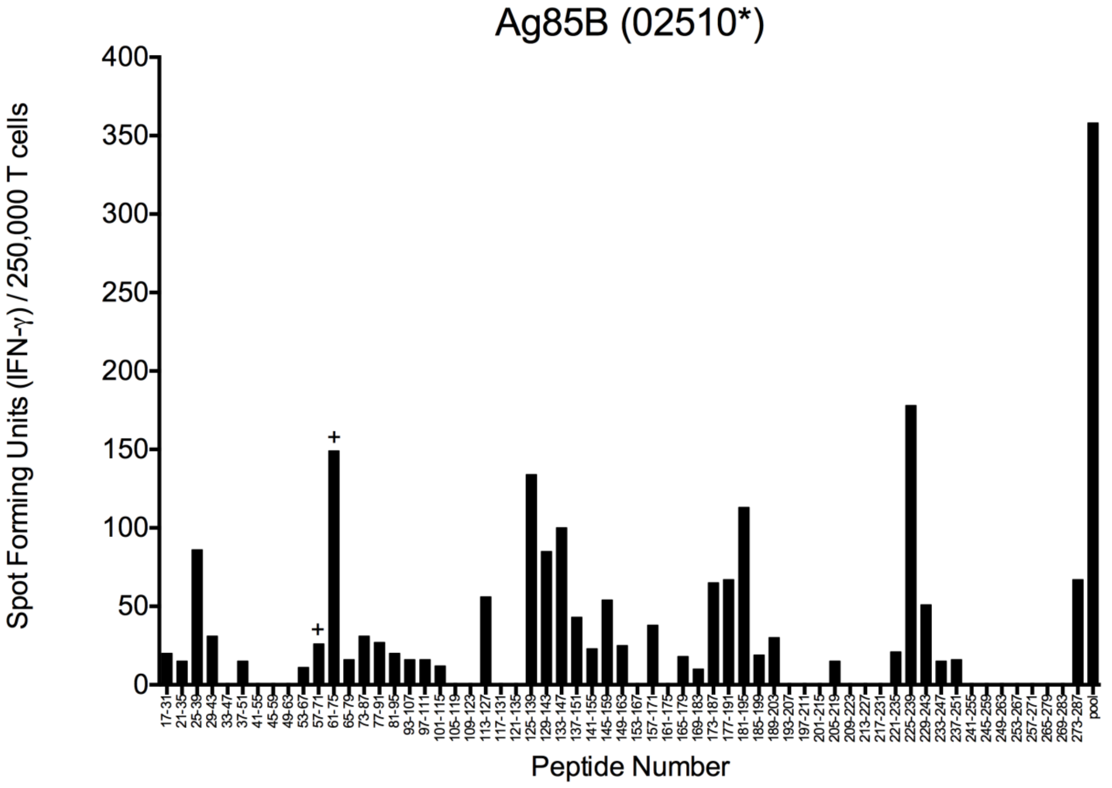
Ag85B (02510*)
Spot Forming Units (IFN-γ) / 250,000 T cells
Peptide Number
0
50
100
150
200
250
300
350
400
+
+
pool

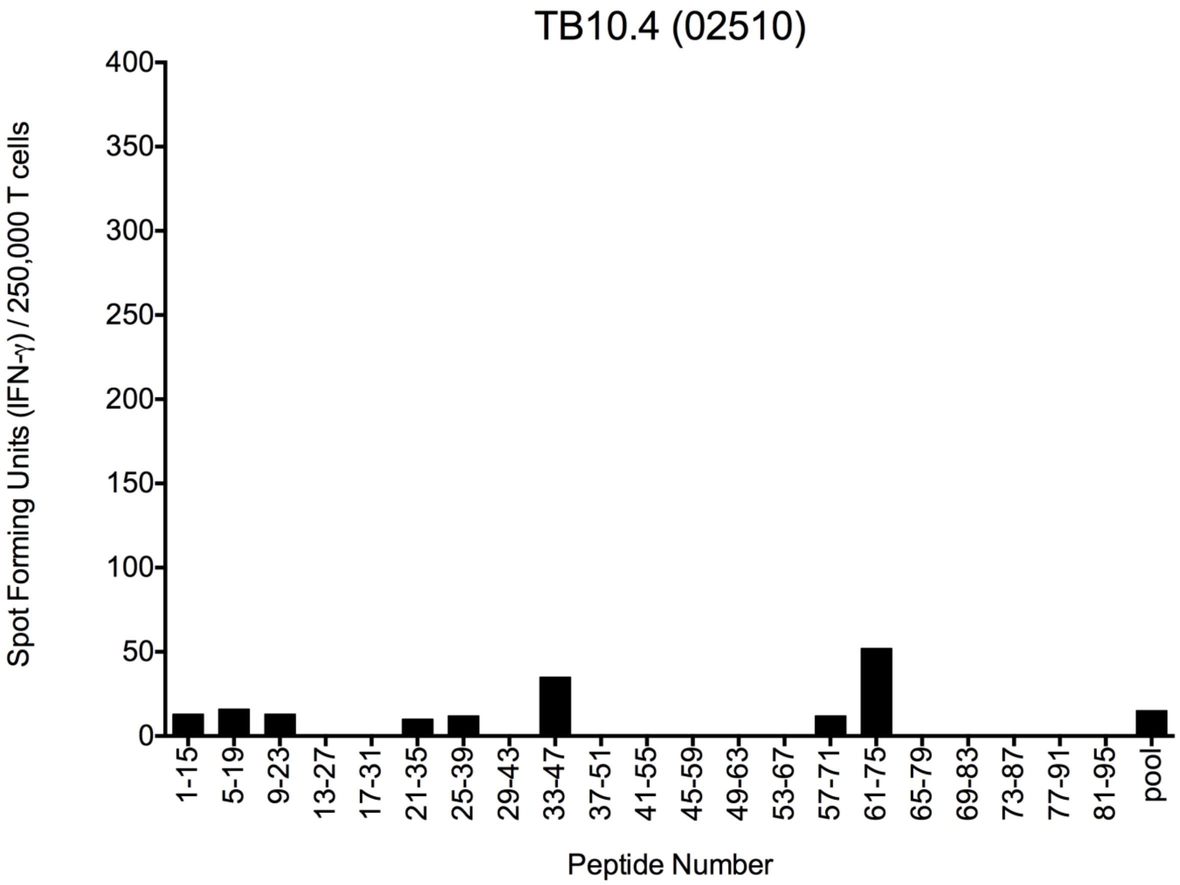
TB10.4 (02510)
Spot Forming Units (IFN-γ) / 250,000 T cells
400
350
300
250
200
150
100
50
0
1-15
5-19
9-23
13-27
17-31
21-35
25-39
29-43
33-47
37-51
41-55
45-59
49-63
53-67
57-71
61-75
65-79
69-83
73-87
77-91
81-95
pool
Peptide Number

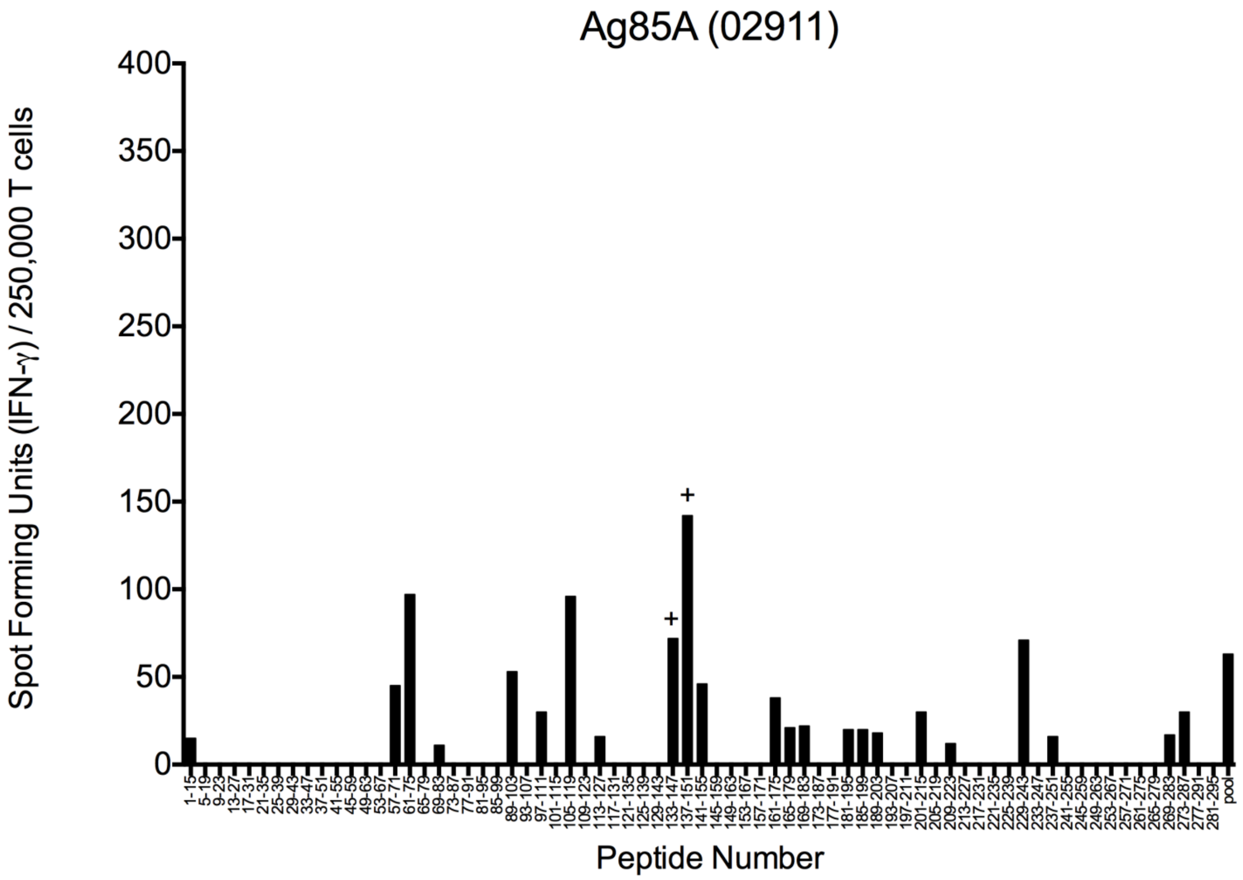

Ag85A (02911)
Spot Forming Units (IFN-γ) / 250,000 T cells
400
350
300
250
200
150
100
50
0
1-15 5-19 9-23 13-27 17-31 21-35 25-39 29-43 33-47 37-51 41-55 45-59 49-63 53-67 57-71 61-75 65-79 69-83 73-87 77-91 81-95 85-99 89-103 93-107 97-111 101-115 105-119 109-123 113-127 117-131 121-135 125-139 129-143 133-147 137-151 141-155 145-159 149-163 153-167 157-171 161-175 165-179 169-183 173-187 177-191 181-195 185-199 189-203 193-207 197-211 201-215 205-219 209-223 213-227 217-231 221-235 225-239 229-243 233-247 237-251 241-255 245-259 249-263 253-267 257-271 261-275 265-279 269-283 273-287 277-291 281-295 pool
Peptide Number

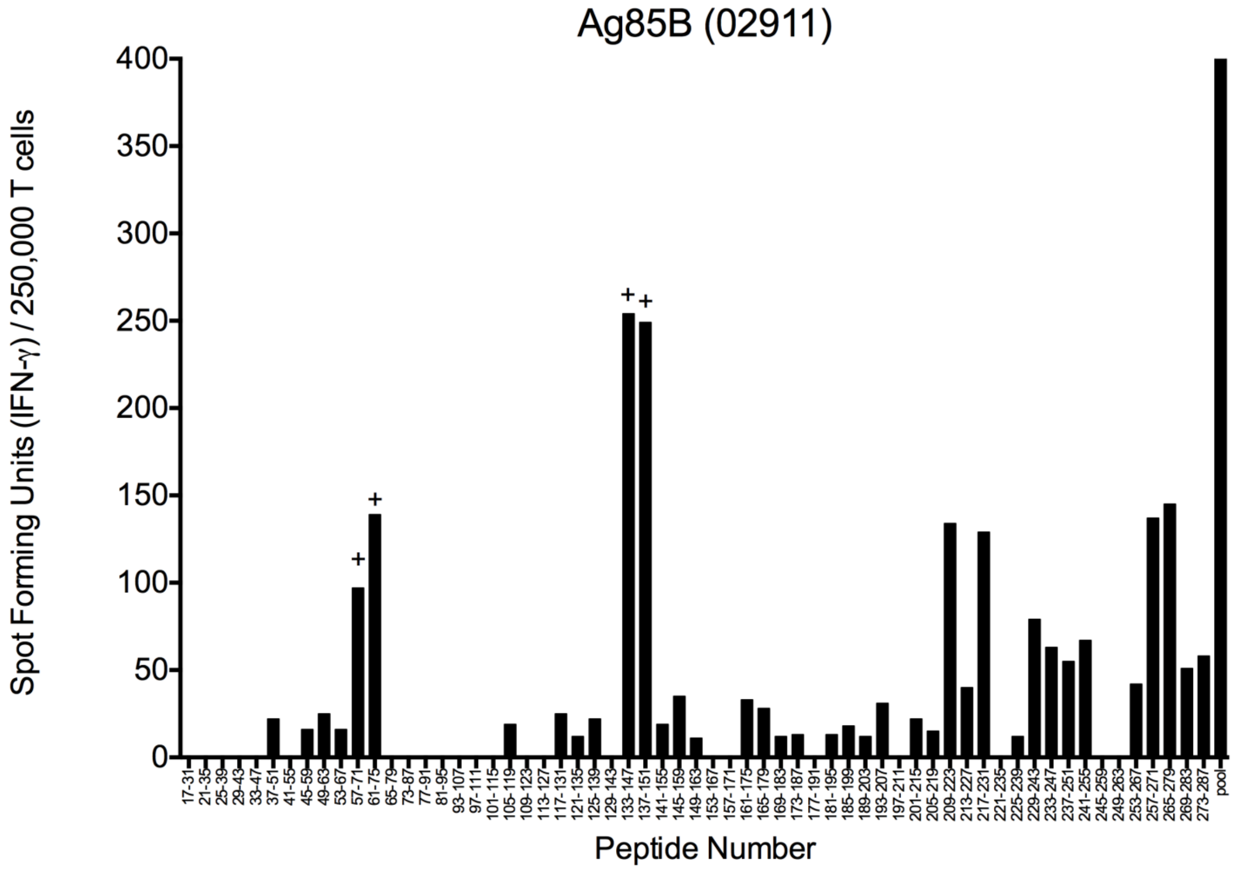

Ag85B (02911)
Spot Forming Units (IFN-γ) / 250,000 T cells
400
350
300
250
200
150
100
50
0
17-31 21-35 25-39 29-43 33-47 37-51 41-55 45-59 49-63 53-67 57-71 61-75 65-79 73-87 77-91 81-95 93-107 97-111 101-115 105-119 109-123 113-127 117-131 121-135 125-139 129-143 133-147 137-151 141-155 145-159 149-163 153-167 157-171 161-175 165-179 169-183 173-187 177-191 181-195 185-199 189-203 193-207 197-211 201-215 205-219 209-223 213-227 217-231 221-235 225-239 229-243 233-247 237-251 241-255 245-259 249-263 253-267 257-271 265-279 269-283 273-287 pool
Peptide Number

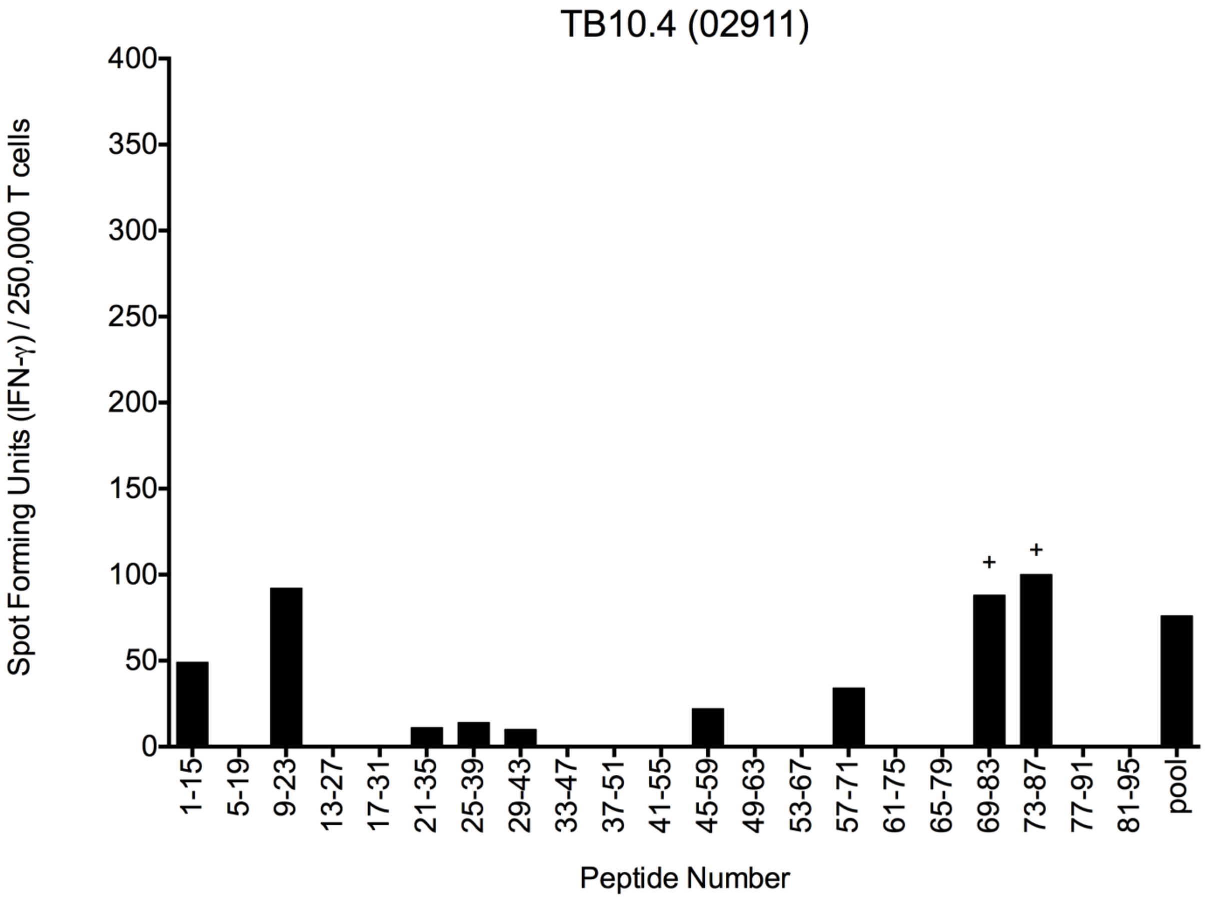

Supplementary Figure S3. Frequency of *ex vivo* CD8⁺ specific responses to 15mer peptides in AERAS-402. To map epitopes recognized by $CD8^+$ T cells to AERAS-402, a participant's *ex vivo* response was determined by IFN-γ ELISPOT in response to all 15mers for the three antigens contained in AERAS-402 (Ag85A, Ag85B, TB10.4) and then with the peptide pool. $CD8^+$ T cells were positively selected from PBMC using magnetic beads (Stemcell Technologies) such that >97% of the cell population were $CD8^+$ T cells. These $CD8^+$ T cells were used as a source of responder T cells and tested in duplicate at a cell concentration of 250,000 cells per well. Autologous DC (20,000 cells/well) were used as APC and peptide pools (5 ug/ml, final concentration of each peptide) were added to the assay. Where sufficient PBMC was not available to make DC, CD4 depleted cells were used (CD8/others*). The antigen is followed by the participant identification in parentheses. Stable $CD8^+$ T cell clones that were derived are illustrated (+) and also summarized in Table 4. For participant 01805, clone A4-2(4) $Ag85B_{65\text{-}79}$ and clone A6-2(0) $Ag85B_{249\text{-}263}$, the Ag85a illustration (±) denotes that we did not have sufficient PBMC to perform ex-vivo deconvolution of the Ag85B peptide pool.

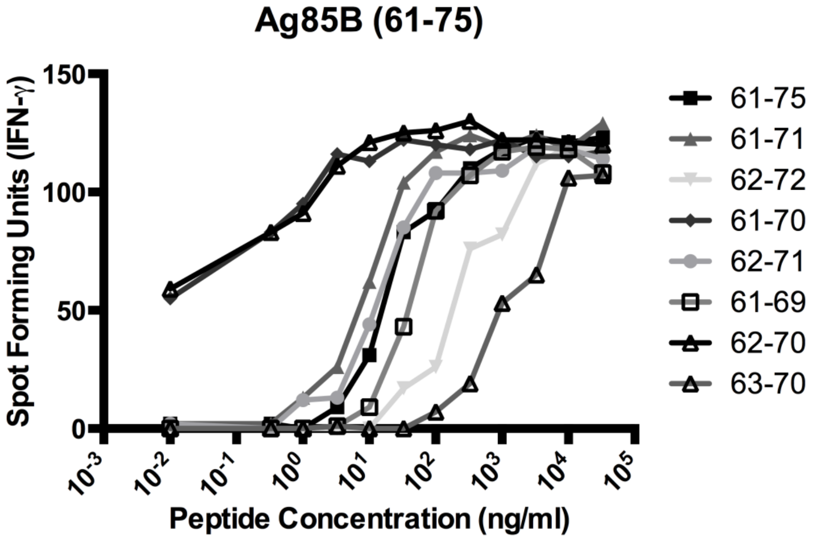

Supplementary Figure S4. Minimal Epitope Titration for Antigen 85B$_{61\text{-}75}$. To map the minimal epitope, autologous LCL (20,000 cells/well) were pulsed with peptide at the concentrations indicated and co-cultured with T cells (1000 cells/well). IFN-γ was assessed by ELISPOT after 18 h co-culture. Each point represents the mean of duplicate determinations. Participant 02911 is shown as a representative example.

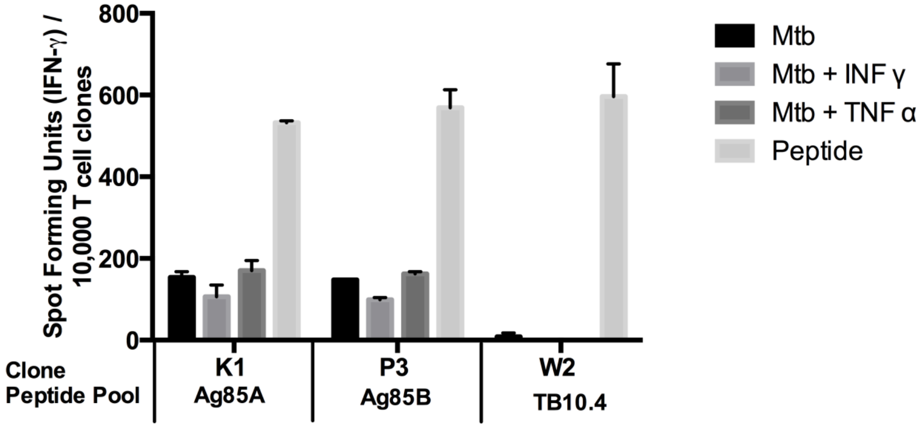

Supplementary Figure S5. Treatment with TNFα and IFN-γ does not increase recognition of Mtb by T cell clones. 10,000 T cell clones were incubated in the presence of 0.5ng/ml IL-2 overnight in an ELISPOT with autologous DC pulsed with 5ug/ml peptide or DC infected (MOI 30:1) with Mtb that had been incubated with IFN-γ, TNF-α or no cytokine. Participant 02911 is shown as a representative example. Error bars represent the standard deviation of the mean of duplicate determinations.

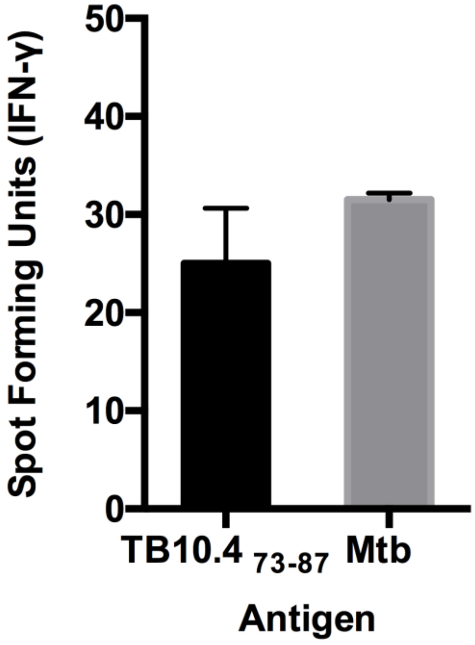

Supplementary Figure S6. A CD8+ T cell clone generated from participant with LTBI (D603W4 TB10.4 $_{73\text{-}87}$) using same cloning method recognizes Mtb-infected DC. 10,000 T cell clones were incubated in the presence of 0.5ng/ml IL-2 overnight in an ELISPOT with autologous DC pulsed with 5ug/ml peptide TB10.4 (aa 73-87; SSTHEANTMAMMARD) or DC infected (MOI 30:1) with Mtb. Error bars represent the standard deviation of the mean of duplicate determinations.

Evaluation	Screen (45 Days)	Study Day											
		-84	-77	-56	-14	0	7	14	28	35	42	56	98
Visit window*	NA	NA	±1	±3	±7	±7	±1	±3	±3	±1	±3	±3	±7
Written informed consent	x												
Assess eligibility criteria	x												
Verify eligibility criteria		x				x			x				
Medical history[h]	x	x	x	x	x	x							
Physical examination[h]	x	x	x	x	x	x							
Serum β-hCG[f]	x												
Vital signs	x	x[c]				x[c]			x[c]				
Urine illicit drug screen	x												
ECG	x												
QuantiFERON	x												x
Hepatitis B, C	x												
HIV-1	x												x
Urinalysis	x[a]					x[a]	x		x[a]	x		x	
Serum chemistry[b]	x[a]					x[a]	x		x[a]	x		x	
CBC, differential, platelets	x[a]					x[a]	x		x[a]	x		x	
Urine β-hCG[f]		x				x			x				
AERAS-402 neutralization						x						x	
HLA						x							
Immunology: ICS**, ELISPOT		x			x				x			x	
Leukapheresis					x								x[d]
**BCG administration**		**x**											
**AERAS-402/placebo administration**						**x**			**x**				
Adverse events (incl con meds)						x	x	x	x	x	x	x	
Serious adverse events (incl con meds)						x	x	x	x	x	x	x	x[e]
Site(s) of injection examination		x	x	x		x	x	x	x	x	x	x	
Phlebotomy by visit, mL[g]	17	40			40	19	7		47	7		54	5
Cumulative phlebotomy volume	17	57			97	116	123		170	177		231	236

NA=Not applicable

*Windows for Study Days -77, -56, -14 and Study Day 0 are based on the date of the Study Day -84 visit. Windows for Study Days 7, 14, 28 and 98 are based on the date of the Study Day 0 visit. Windows for Study Days 35, 42 and 56 are based on the date of the Study Day 28 visit.

** ICS was also performed on day 98.

a. These screening evaluations were done no earlier than 7 days prior to BCG administration on Study Day -84 and 3 days prior to study vaccine administration on Study Day 0 and Study Day 28.
b. Serum chemistries = ALT, AST, total bilirubin, ALP and creatinine.
c. Vital signs (blood pressure, heart rate, oral temperature) obtained pre-vaccination, and 30±5 and 60±5 minutes post-vaccination.
d. Study Day 98 leukapheresis was performed any time after the Study Day 56 visit but no later than the Study Day 98 visit.
e. Follow-up for serious adverse events is continued by 6-monthly telephone contact as part of the Registry Protocol.
f. For females of child-bearing potential only. Serum β-hCG was done no earlier than 7 days prior to Study Day -84. Urine β-hCG was done on Study Day -84 prior to BCG administration and on Study Day 0 and Study Day 28 prior to study vaccine administration.
g. Phlebotomy volumes were based on the following estimates: Serum β-hCG: 1 mL, QuantiFERON: 3 mL, Hepatitis B/C: 4 mL, HIV-1: 2 mL, Chemistry: 2 mL, CBC: 5 mL, AERAS-402 neutralization: 7 mL, HLA: 5 mL, ICS: 24 mL, ELISPOT: 16 mL.
h. Update medical history and physical examination from Study Days -84 through Study Day 0.

Note: The amount of blood required for this study is within WHO guidelines for blood donation.

Supplementary Table S1. Complete Summary Schedule of Participant Evaluations
